# Potential Role of Notch Signalling in CD34^+^ Chronic Myeloid Leukaemia Cells: Cross-Talk between Notch and BCR-ABL

**DOI:** 10.1371/journal.pone.0123016

**Published:** 2015-04-07

**Authors:** Abdullah Aljedai, Anne-Marie Buckle, Prashant Hiwarkar, Farhatullah Syed

**Affiliations:** 1 Faculty of Life Sciences, Manchester Institute of Biotechnology (MIB), University of Manchester, Manchester, United Kingdom; 2 Department of Paediatric Haematology, Great Ormond Street Hospital (GOSH), London, United Kingdom; 3 Institute of Inflammation and Repair, Manchester Institute of Biotechnology (MIB), University of Manchester, Manchester, United Kingdom; Università degli Studi di Firenze, ITALY

## Abstract

Notch signalling is critical for haemopoietic stem cell (HSC) self-renewal and survival. The role of Notch signalling has been reported recently in chronic myeloid leukaemia (CML) – a stem cell disease characterized by BCR-ABL tyrosine kinase activation. Therefore, we studied the relationship between BCR-ABL and Notch signalling and assessed the expression patterns of Notch and its downstream target *Hes1* in CD34^+^ stem and progenitor cells from chronic-phase CML patients and bone marrow (BM) from normal subjects (NBM). We found significant upregulation (*p<0*.*05*) of *Notch1*, *Notch2* and *Hes1* on the most primitive CD34^+^Thy^+^ subset of CML CD34^+^ cells suggesting that active Notch signalling in CML primitive progenitors. In addition, Notch1 was also expressed in distinct lymphoid and myeloid progenitors within the CD34^+^ population of primary CML cells. To further delineate the possible role and interactions of Notch with BCR-ABL in CD34^+^ primary cells from chronic-phase CML, we used P-crkl detection as a surrogate assay of BCR-ABL tyrosine kinase activity. Our data revealed that Imatinib (IM) induced BCR-ABL inhibition results in significant (*p<0*.*05*) upregulation of Notch activity, assessed by *Hes1* expression. Similarly, inhibition of Notch leads to hyperactivation of BCR-ABL. This antagonistic relationship between Notch and BCR-ABL signalling was confirmed in K562 and ALL-SIL cell lines. In K562, we further validated this antagonistic relationship by inhibiting histone deacetylase (HDAC) - an effector pathway of *Hes1*, using valproic acid (VPA) - a HDAC inhibitor. Finally, we also confirmed the potential antagonism between Notch and BCR/ABL in *In Vivo*, using publically available GSE-database, by analysing gene expression profile of paired samples from chronic-phase CML patients pre- and post-Imatinib therapy. Thus, we have demonstrated an antagonistic relationship between Notch and BCR-ABL in CML. A combined inhibition of Notch and BCR-ABL may therefore provide superior clinical response over tyrosine-kinase inhibitor monotherapy by targeting both quiescent leukaemic stem cells and differentiated leukaemic cells and hence must be explored.

## Introduction

The Notch signalling is evolutionarily conserved pathway, which plays an important role in regulating the process of development [1,2]. Notch signalling is composed of a Notch receptor, ligand and CBF1/Su(H)/Lag-1 family (CSL) DNA binding protein. In mammals, four Notch receptors (*Notch1-4*) and five Notch ligands (*Jagged1* and *2*, and *Delta-like1*, *3* and *4*) have been identified [3]. Upon binding of Notch ligands to its receptors, the receptor undergoes at least two proteolytic cleavages, releasing the activated Notch intracellular domain (NICD) into the cytoplasm, which then translocate to the nucleus and subsequently activates the transcription of the Notch target genes. The important downstream target genes of Notch include *Hes1*, *Hes5* and *Hey*. The activated Notch pathway determines the cell fate and maintains the stem cell state [1,2].

Chronic myeloid leukaemia (CML) is a stem cell disease with the differentiated cells in CML constituting the bulk of leukemic cell mass, whereas the leukemic stem cells are responsible for the disease maintenance [4,5]. It has been shown that IM is highly toxic to the differentiated CML progenitors but not to the leukemic stem cells which remain viable in a quiescent state [6,7]. Therefore, it is possible that CML stem cells survival and self-renewal capacity is related to the same pathway, which regulates the normal HSCs such as Notch signalling pathway.

Well established role for Notch signalling in human T-cell acute lymphoblastic leukaemia (T-ALL) [8,9], and the axon development in *Drosophila* support the hypothesis of possible interactions between ABL protein kinase and Notch signalling. It has been found that *Notch* interacts genetically with *ABL*, as *Notch* and *ABL* mutations synergise to cause synthetic lethality in *Drosophila* axons [10]. In another study, Giniger and colleagues have also found that Delta ligand and Notch provides a guidance signal to the developing axon by regulating the ABL kinase signalling pathway [11].

In CML, Notch signalling has been demonstrated to mediate the disease progression [12] and in K562 CML cell line model Notch signalling inhibited the development of erythroid/megakaryocytic cells by induction of *Hes1* [13] and proliferation of K562 cells [14]. Recently, Yang *et al*., showed that over-expression of *Notch2* inhibits the proliferation of CML cells [15,16]. *Hes1* which is the most widely characterised Notch downstream target gene has been shown to immortalize committed progenitors and play a role in transformation of chronic-phase CML to blast crisis [9,17,18]. However, the underlying molecular relationship between Notch signalling and CML remains largely unknown. Based on the above findings and the notion that Notch co-operates with several signal-transduction pathways to induce leukaemogenesis, we hypothesized that Notch signalling may be altered in CML, and that Notch might interact with the BCR-ABL fusion protein in CML.

Therefore based on our hypothesis the objectives of this study were (i) To investigate the expression of Notch receptors in CD34+ primary CML at mRNA and protein level, (ii) To investigate the expression of Notch target genes *Hes1* and *Herp1&2* using PCR to determine the activity of Notch signalling in CD34+ primary CML cells, (iii) To investigate the possible cross-talk between Notch and BCR-ABL in primary CD34+ CML cells as well as in cell line models, and lastly (iv) To validate the Notch-BCR-ABL relationship using CML microarray datasets from GSE database.

## Materials and Methods

### Primary chronic myeloid leukaemia samples

Fresh or frozen peripheral blood samples from non-treated patients with chronic myeloid leukaemia (CML) in chronic phase were used in this project. Bone marrow (BM) of normal subjects (NBM) and cord blood from normal subjects were used as controls.

### Ethics statement

Bone marrow and cord blood samples were kindly provided by Dr. John Burthem (Clinical Senior Lecturer, University of Manchester, Manchester, UK) and all the samples were ethically approved by University of Manchester committee as described [19]. CML samples were kindly provided by Prof., Tessa Holyoake, (professor of Experimental Hematology, Paul O'Gorman Research Centre, Gartnavel General Hospital, Glasgow, UK) and were ethically approved [20,21].

### Isolation of mononuclear cells (MNC)

Mononuclear cells from blood samples were isolated using ficoll-paque (Amersham Pharmacia Biotech, UK) density gradient method under sterile conditions according to the manufacturer’s instructions. Samples were diluted 1:1 with hanks balanced salt solution (HBSS) (Sigma-Aldrich, UK) supplemented with 5% newborn calf serum (NCS) (Invitrogen, UK). 20 ml of the diluted blood was then carefully layered onto 10 ml ficoll in a 50 ml falcon tube and centrifuged at 389g for 30 minutes at room temperature (RT). Mononuclear cells were harvested from the interface layer and washed twice with 50 ml HBSS/5%NCS by centrifugation at 389g at RT for 7 minutes. The pellet was then re-suspended in known volume of HBSS/5% NCS for FACS sorting, or processed for storage in liquid nitrogen.

### Isolation of haemopoietic progenitor cell populations

Haemopoietic progenitors positive for CD34 were isolated from cord blood from normal subjects and CML sample, using StemSep kit (StemCell Technologies, UK) according to the manufacturers’ instructions. The eluted CD34+ve cells were then pooled and viability assessed before cells were pelleted and re-suspended in 100 μl of (1:20 dilution) CD34-APC, (1:20 dilution) Thy-PE and (1:20 dilution) Lin-FITC cocktail and incubated for 20 minutes at 4°C in the dark. Cells were then washed with 2 ml HBSS/5% serum and re-suspended in 1 ml HBSS/5% serum for sorting. Cells were sorted into a 24 well plate using a FACS Vantage (Becton Dickinson, USA) flow cytometer. Sorted cells were then transferred into RNAse free eppendorf tubes.

### Cell lines used in this study

K562 (ATCC, CCL243), SIL-ALL [22,23], CEM [22,23] and JURKAT (ATCC, TIB152) cells were maintained in RPMI 1640 (Sigma-Aldrich, UK) supplemented with 10% (v/v) fetal bovine serum (FBS) (Sigma-Aldrich, UK), 2 mM L-glutamine and 0.1 mg/ml penicillin/streptomycin (Sigma-Aldrich, UK) at 37°C within 5% CO2. Cell lines were sub-cultured every 3–4 days and transferred to fresh media to maintain long phase growth. All the cell lines used in this study were below passage-20.

### Short-term liquid culture of primary CML CD34+ cells

CD34+ cells were cultured in serum free expansion medium (SFEM) (StemCell Technologies, UK) supplemented with 1% glutamine (100 mM) and 1% penicillin-streptomycin (100 mM) (Invitrogen, UK). SFEM was further supplemented with growth factor cocktail comprising 100 ng/ml Flt3- ligand, 100 ng/ml stem cell factor, 20 ng/ml each of interleukin (IL)-3, IL-6 and granulocyte colony stimulating factor (GCSF) (R&D Systems, UK).

### FACS analysis of extra- and intra-cellular Notch1

To study the expression of the extra-cellular Notch1 (ECN1) and intra-cellular Notch1 (ICN1) on the cell surface of K562 /primary CD34+ CML, cells (1x10^6^) were directly stained with EA1 and b-TAN20 antibody respectively (S1 Table) as described previously [24,25]. As K562 cells are negative for CD34 surface antigen the analysis gate used here included all live K562 cells.

### Poly-A PCR

RNA extraction, construction of cDNA from low cell number, cDNA tailing reaction and poly-A PCR was carried out as described previously [22].

#### Construction of cDNA from high cell numbers

High Capacity cDNA Reverse Transcription Archive Kit (Applied Biosystems, UK) was used for the cDNA synthesis from cell numbers higher than 1x10^5^ cells, according to the manufacturer’s instructions.

#### Gene specific PCR

Gene specific PCR was performed in 10 μl reactions consisting of 5 μl PCR Reddy Mix (ABgene, UK), 0.5 μl of forward and reverse primers, (S2 Table) (Sigma-Aldrich, UK) and 1 μl of human genomic DNA diluted 1:500 (Promega, UK) as described previously [22]. Agarose gel electrophoresis was used to resolve and visualise PCR product using 1.5% agarose (Sigma-Aldrich, UK) in TBE. The size of the PCR products was determined by the GeneRuler 100bp ladder (Fermentas, UK). Finally, the resulting gel was observed on a Typhon 8600.

### QRT-PCR using TaqMan probes

Real time PCR experiments were performed using TaqMan probes as described previously [22,25]. This method was used to measure *Notch1*, *Notch2*, and *Hes1* in CML patients as well as bone marrow from normal subjects. S3 Table shows the list of primers and TaqMan probes used in real time PCR.

### The P-crkl assay

CRKL is an important substrate of the BCR-ABL oncoprotein in CML and binds to both BCR-ABL and c-Abl.[26] Crkl tyrosine is phosphorylated in CML cells which is absent in normal haemopoietic cells,[26] hence it has been recognized as a prognostic marker for CML. The levels of phosphorylated crkl (P-crkl) were measured by intra-cellular FACS. Cells (1 x 10^5^) were harvested and washed once in HBBS/5% FBS, then fixed in fixing reagent (Caltag Laboratories, UK) and incubated at RT for 15 minutes. The cells were then washed once with 3 ml HBBS/ 5% FBS and re-suspended with 25 μl permeabilizing reagent (Caltag Laboratories, UK) and 2.5 μl of P-crkl primary antibody (New England Biolabs, UK). The cells were then gently vortexed and incubated at RT for 40 minutes, before being washed twice with 3 ml HBBS/ 5% FBS. The secondary antibody was added directly at the appropriate dilution and the cells were mixed and incubated at RT for 30 minutes in the dark. The P-crkl results were reported as mean fluorescence intensity (MFI).

### Western blotting

Total protein was extracted from K562 cells using RIPA buffer (Sigma-Aldrich, UK) containing phosphatase and protease inhibitors (Sigma-Aldrich, UK). Protein concentration was determined by bio-rad protein assay (Bio-Rad, UK). Proteins were separated on SDS-PAGE and separated proteins were transferred to a nitrocellulose membrane (Sigma-Aldrich, UK). The membrane was incubated in blocking buffer (5% skimmed milk in 1x PBS containing1% (v/v) TWEEN 20) (Sigma-Aldrich, UK) for 1 hr at RT and subsequently stained with primary antibody (S2 Table) for 2h at RT or at 4°C overnight. The membrane was then washed once with water and three times with PBST (1x PBS containing 1% (v/v) TWEEN 20) for 15 minutes at RT prior to adding secondary antibody for one hour at RT. The membrane was washed 4 times at 10 minutes intervals at RT. After applying chemiluminescence substrate (Pierce, UK), the membrane was developed using an autoradiography in a Fuji film FPM800A automated developer.

### Analysis of CML CD34+ patients and BM from normal subjects microarray dataset from GSE database

Three datasets of microarrays on CD34+ cells isolated from BM from normal subjects and patients with CML were retrieved from GSE database. These datasets were GSE550 (n = 17; Affymetrix Human HG-Focus Target Array), GSE1418 (n = 14; Affymetrix Human HG-Focus Target Array), GSE12211 (n = 12; Affymetrix Human Genome U133A 2.0 Array) [27–29]. 7947 genes common to these three datasets were used for analysis. Using Combat function in R, batch effects in these datasets were combatted as described previously [30]. In these datasets, there were normal donors (n = 14), CML patients prior treatment (n = 15), CML patients treated with Imatinib (IM) for a median duration of 28 months (n = 8) and CML patients treated with IM for 7 days (n = 6). Three axis principal component analysis (PCA) was performed on combatted datasets using DUDI.PCA function. Gene set enrichment analysis (GSEA) as previously described, using KEGG pathways was also performed [31]. Gene expression signature of BM from normal subjects was compared with the paired samples from GSE12211 datasets which included samples from patients with CML pre- and 7 days post-IM treatment.

### Statistical analysis of datasets

The Mann-Whitney test was used to compare the differences in gene expression between BM from normal subjects and CML biological samples. To assess the statistical difference in drug treated samples, a paired T-test was carried out. All differences with *p<0*.*05* were considered statistically significant. Data were expressed as mean± SEM for all the statistical analysis.

## Results

### Expression profile of Notch receptors and Notch target genes in CD34+ cells in the chronic phase of chronic myeloid leukaemia at transcriptional level

**Fig 1:**
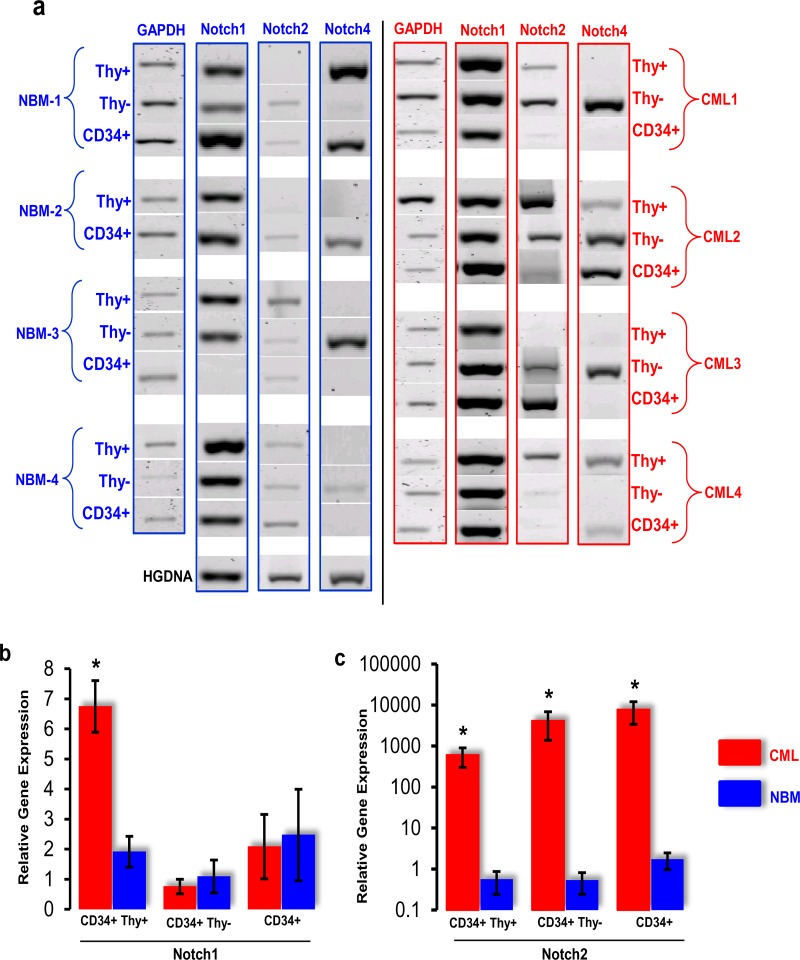
Expression of *Notch* receptor in CD34+ cells isolated from BM of normal subjects and CML patients. **(a)** Conventional PCR products are shown for four bone marrow (NBM1-4) samples from normal subjects on the left panel and for four CML samples on the right panel. For each sample, the expression of *Notch1*, *Notch2*, and *Notch4* within the CD34+, Thy-, Thy+ subpopulation is shown. The housekeeping gene *GAPDH* was used as a control to assess the quality of cDNA in each sample. The lower left panel shows human genomic DNA (HGDNA) as a positive control for each set of oligonucleotides. Bar graph shows real-time PCR analysis of *Notch1* **(b)** and *Notch2* **(c)** expression on CD34+ subsets from NBM and CML patients. Gene expression was normalised to the *GAPDH*. *Notch1* showed significant upregulation in the CD34+ Thy+ cell subset (*p<0*.*05*). *Notch2* showed significant (*p<0*.*05*) upregulation in all the CD34+ CML primary subset cells compared with NBM.

The expression profile of *Notch1–4* receptors was studied in four CML patient samples along with four bone marrow samples from normal subjects (NBM) using the polyA PCR technique. Cells in each sample were fractionated into CD34+ Thy+, CD34+ Thy-, and total CD34+ subsets to enable the study of gene expression in haemopoietic progenitors at different maturation levels and sorted cells were of 95% purity. Fig 1a shows the PCR profile of *Notch* receptors in both NBM and CML samples. The housekeeping gene *GAPDH* was used to assess the quality of cDNA and to check the uniformity of DNA content among different samples.

*Notch1* was expressed in all the normal samples (n = 4) and in all three haemopoietic CD34+, Thy+, and Thy- subpopulations (n = 4). Interestingly, Thy+ and Thy-, both subsets expressed *Notch1* in the CD34+ cells taken from BM of normal subjects. Similar results were also seen in CML samples with no clear evidence of differences in the expression between the CD34+, Thy+, and Thy- subpopulations. *Notch2* was weakly expressed in all three CD34+ subpopulations in both NBM and CML samples. *Notch*3 did not show any expression in either NBM or CML samples (data not shown). *Notch4* was irregularly expressed and was seen in two normal and two CML CD34+ samples, respectively (Fig 1a).

In order to determine the significant difference between the *Notch* expression seen in NBM and CML CD34+ populations, quantitative RT-PCR was performed. QRT-PCR showed a 3-4-fold statistically significant (*p*<0.05) upregulation in *Notch1* transcripts in CD34+ Thy+ cell subset compared with NBM. However, no significant difference was observed in *Notch1* expression in CD34+ CML and CD34+ Thy- subpopulation compared to NBM (Fig 1b). Interestingly, *Notch2* was significantly (*p* = 0.02) upregulated in all CML subpopulations (n = 4) compared with NBM. There was more than a 100-fold increase in *Notch2* expression in the CD34+ Thy+, CD34+ Thy-, and in the total CD34+ cell subsets as compared with NBM samples (Fig 1c).

**Fig 2:**
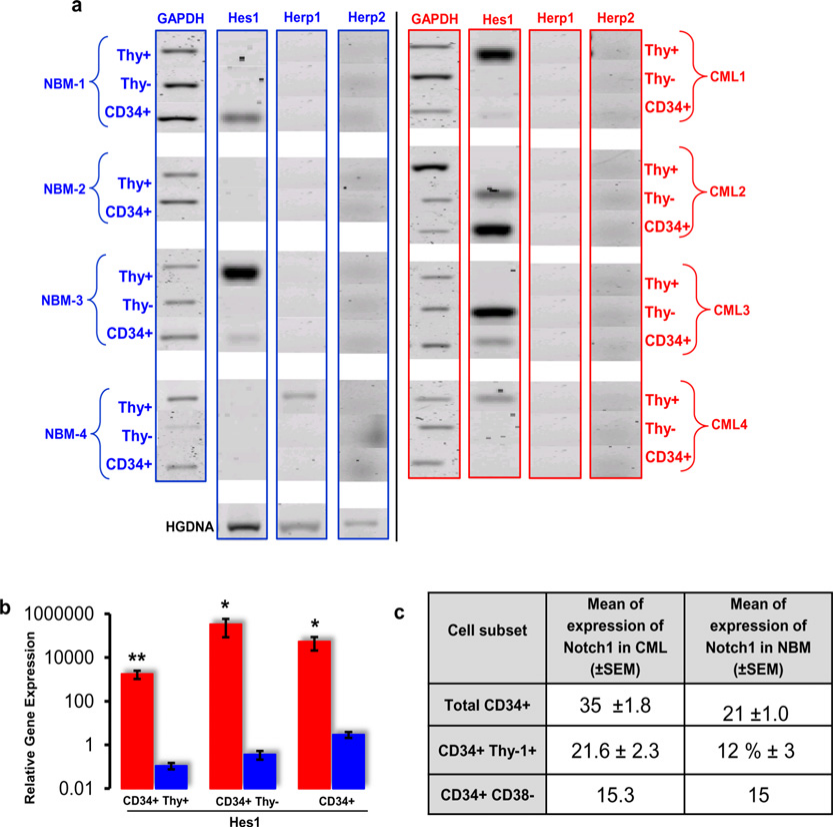
Expression of *Notch* receptors and its target genes in CD34+ cells isolated from BM of normal subjects and CML patients. **(a)** Conventional PCR products are shown for four NBM (left panel) and four CML samples (right panel). *GAPDH* was used to assess the quality of cDNA. The lower left panel shows human genomic DNA (HGDNA) as a positive control for each set of oligonucleotides. (**b)** Real-time PCR analysis of *Hes1* expression on CD34+ cell subsets from NBM and CML patients. Results showed significant (* = *p<0*.*05*, ** = *p≤0*.*01*) upregulation of *Hes1* in all the CD34+ CML primary subset cells compared with NBM. **(c)** Summary of Notch1 expression profile in different cell lineages in CML and NBM. FACS analysis of Notch1 in different myeloid, lymphoid, and more primitive lineages in CML was done by co-staining mononuclear cells with both extracellular Notch1 (ECN1-EA1) antibody and a lineage-specific cell surface marker. Results shown here are representative of the total CD34+ cells in each sample. The mean of expression refers to the percentage of each cell population in the left column that was positive for EA1. The means of expression were measured from four different CML samples (n = 4).

The expression of Notch target genes *Hes1*, *Herp1*, and *Herp2* was also studied to assess the active Notch signalling in CML compared with normal CD34+ cells. Results showed that neither *Herp1* nor *Herp2* was expressed in both NBM and CML patient samples. Interestingly, *Hes1* expression was seen in both normal and CML samples with no precise pattern of activity discernible, suggesting that Notch signalling was activated in these samples (Fig 2a). However, quantitative real-time PCR analysis demonstrated a greater than 100-fold significant (*p<0*.*05*) increase in the *Hes1* expression in all the CML CD34+ cell subsets (n = 4) compared with NBM (n = 4) (Fig 2b).

### Differential expression profile of Notch1 in chronic myeloid leukaemia at protein level

Does overexpression of *Notch1* mRNA get translated to its protein product in CML patients? To address this question, mononuclear cells from chronic phase CML patients were stained with EA1, a monoclonal antibody [24] that recognises the extracellular domain of *Notch1* (ECN1) [25] (S1 Fig).

In CML, *Notch1* was expressed in 35% of gated CD34+ primary cells compared with 21% of CD34+ cells in normal cord blood (Fig 2c and S2 Fig). Finally, the expression of *Notch1* was also confirmed on the very primitive haemopoietic CD34+ Thy+ and stem cell-enriched CD34+/CD38- progenitor cells, and results showed that *Notch1* was expressed in 21.6 ± 2.3% of the CD34+ Thy+ population and 15.0 ± 0% of the CD4+ CD38- population (n = 4) (Fig 2c and S3 Fig). Interestingly, stem cell-enriched CD34+/CD38- progenitor cells (corresponding to primitive HSC) do not show significant difference in Notch1 expression at protein level between cord blood samples from normal donors and in CML samples from the chronic CML patients (Fig 2c). In conclusion, the above results confirm that *Notch1* is upregulated in the most primitive CD34+ Thy+ cells in the chronic phase of CML as compared to normal cord blood, suggesting that Notch signalling might be involved in the survival and/or self-renewal of leukemic stem cells in CML.

### Demonstration of cross-talk between Notch and BCR-ABL in primary CD34+ chronic myeloid cells in vitro

The above results confirm the overexpression of Notch and its target gene *Hes1* in primary CD34+ CML cells. Moreover, it has been demonstrated that enhanced kinase activity of BCR-ABL and altered expression of *Notch1* synergises to induce acute leukaemia in a transgenic model for CML [32]. Therefore, we initially assessed the interaction between Notch and BCR-ABL in primary CD34+ CML cells and then proceeded to establish these interactions in CML cell line models.

#### (i) Imatinib mesylate (IM) inhibits BCR-ABL activity in chronic phase CML CD34+ primary cells

After establishing the cross-talk between Notch and BCR-ABL in the CML cell line models, we wished to delineate the interaction between Notch and BCR-ABL in the primary CD34+ CML patient cells.

**Fig 3:**
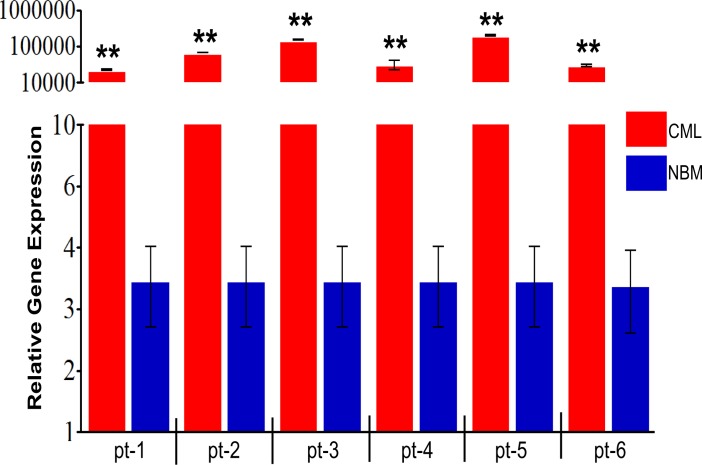
*Hes1* gene expression profile in CD34+ cells isolated from CML patients. The expression profiles of the Notch target gene *Hes1* was investigated by real-time PCR. Data is shown from CD34+ cells isolated from six CML patients (pt) in chronic phase and CD34+ control cells from three NBM samples. Relative gene expression was calculated using the DDCt method. ** = p<0.01; significant expression of *Hes1* in each CML sample was compared with *Hes1* expression in the NBM samples.

Increased kinase activity of BCR-ABL in CD34+ CML primary cells was first confirmed by a P-crkl expression using P-crkl-FITC conjugated antibody (S4 Fig). The CD34+ CML primary cells were isolated from frozen blood samples of CML patients (n = 6) whose Notch activity was not confirmed. Therefore, to assess the activity of Notch signalling in these cells, *Hes1* gene expression was measured in the CD34+ cells in all CML samples by real-time PCR. Results showed that *Hes1* expression was significantly (*p*<0.01) upregulated in CD34+ cells in all six CML samples compared with control (normal CD34+ cells from NBM) group (Fig 3).

**Fig 4:**
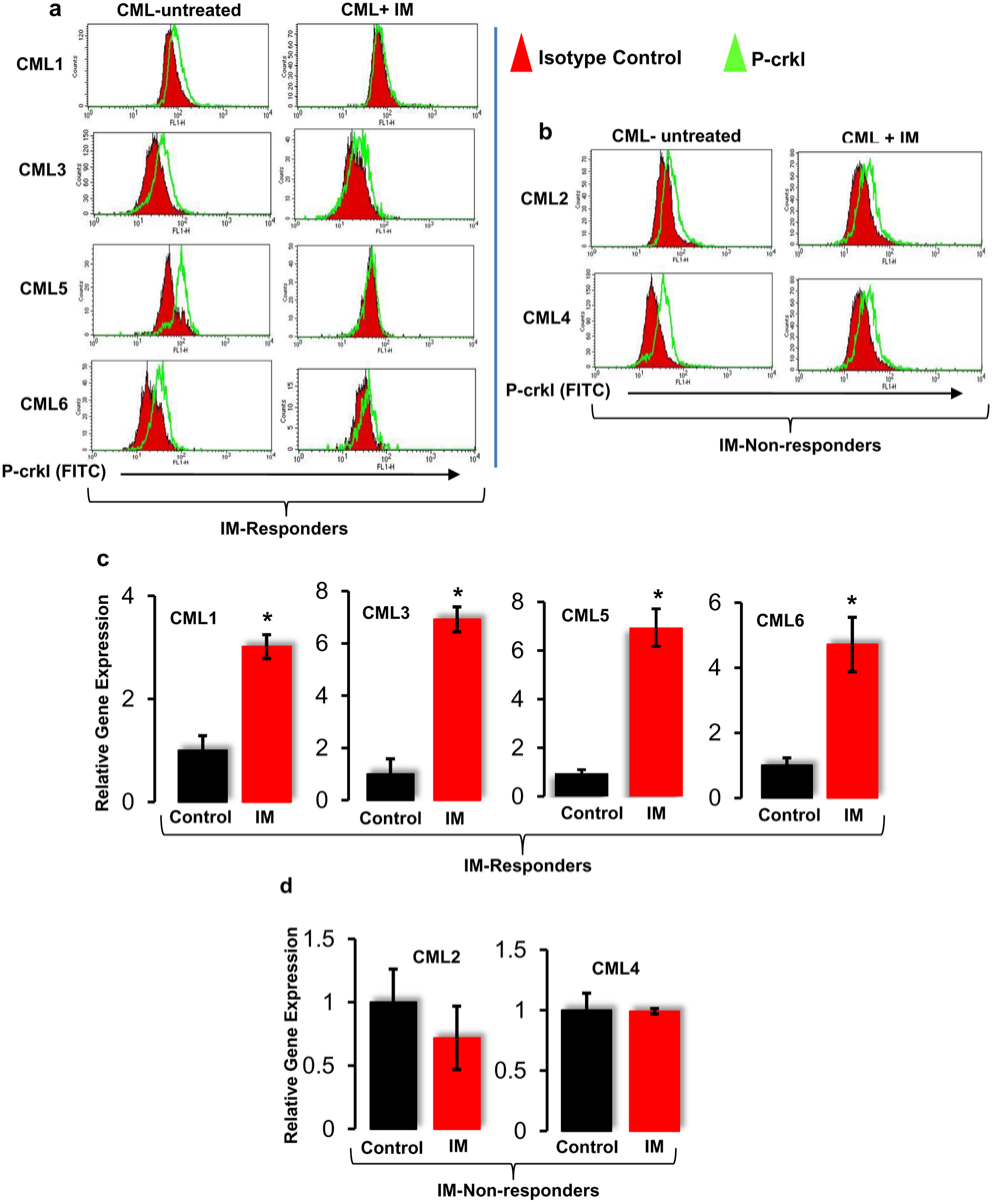
Evaluation of BCR-ABL activity in primary CD34+ CML cells. **(a)** Inhibition of BCR-ABL activity by IM in CD34+ primary cells isolated from CML patients. CD34+ primary cells were cultured in the presence of 10 μM IM for 72h and then stained for P-crkl expression. **(b)** Primary CD34+ cells from two CML patients show resistance to IM, assessed by BCR-ABL activity. **(c)** *Hes1* gene expression in IM-sensitive CD34+ primary CML cells (*p<0.*01*). **(d)** *Hes1* gene expression in CD34+ cells isolated from IM resistant CML patients.

The efficacy of IM on primary CD34+ CML was tested by FACS-based P-crkl assay. Samples from patients with chronic phase CML (n = 5) were enriched for CD34+ and cultured for 24h in serum-free medium (SFM) supplemented with growth factor cocktail comprising 100 ng/mL Flt3-ligand, 100 ng/mL stem cell factor, and 20 ng/mL each of interleukin (IL)-3, IL-6 and granulocyte-colony stimulating factor (G-CSF). Primary CD34+ CML cells were then treated with 10 μM IM for 72h, and the inhibitory effect of IM on CD34+ CML primary cells was assessed by the P-crkl assay, and K562 cells were used as a positive control. Results showed that expression of P-crkl was clearly reduced in CD34+ cells from three CML samples as compared with the control group (Fig 4a). The results also demonstrated that BCR-ABL activity was inhibited by IM in CD34+ cells by marked reduction of crkl phosphorylation. These results are in good agreement with Chu *et al*. (2004), who showed that IM-induced inhibition of crkl phosphorylation in CML CD34+ cells in a dose-dependent manner [33]. However, two out of five CD34+ CML primary cells showed some degree of P-crkl expression at 72h post-IM treatment (Fig 4b), suggesting that these two samples (CML-2 and CML-4) are resistant to IM treatment.

#### (ii) IM upregulated Notch target gene *Hes1* expression in CD34+ CML cells

Next, we investigated the effect of BCR-ABL inhibition on Notch signalling in CD34+ CML primary cells. All CML samples were enriched for CD34+ cells using magnetic CD34 selection. After culturing in the presence of IM, the percentage of CD34 cells was assessed by FACS, and RNA was extracted directly from cultured cells if they were >90% CD34+, or FACS sorted if they were <90% CD34+. The Notch target gene *Hes1* transcript levels were measured using qRT-PCR. There was a significant (*p*<0.05) ~4-fold increase (n = 4) in *Hes1* gene expression following IM treatment (Fig 4c). Interestingly, two CML samples (CML-2 and CML-4), which exhibited resistance to IM, revealed no significant (*p*>0.05) difference in *Hes1* expression compared with the control group (Fig 4d). From the above results, it was concluded that CD34+ CML primary cells sensitive to IM showed further activation of Notch.

#### (iii) GSI induced inhibition of Notch signalling in CD34+ CML cells

**Fig 5:**
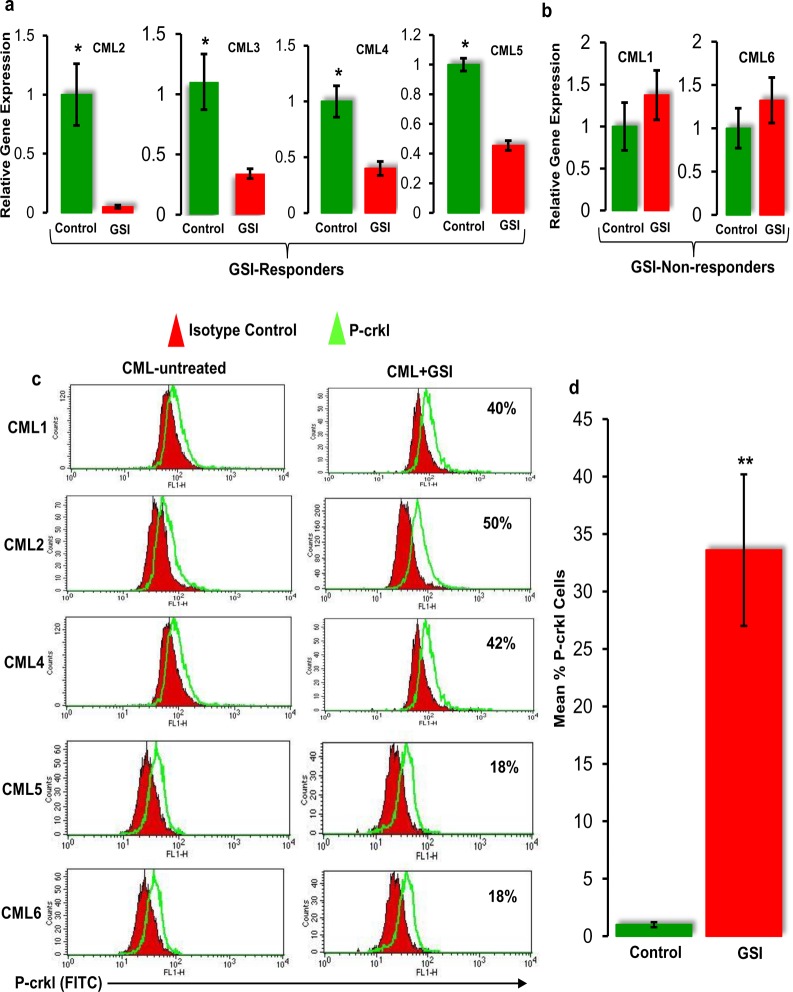
Assessment of cross-talk between Notch and BCR-ABL activity in CD34+ primary CML cells. **(a)** *Hes1* gene expression in CD34+ cells isolated from GSI-responder CML patients. CD34+ cells were isolated from CML patients and cultured in the presence of 10 μM GSI for 72h. Live CD34+ cells were then sorted, and the gene expression of the Notch target gene *Hes1* was investigated by real-time PCR (*p<0.*01*). **(b)** *Hes1* gene expression in CD34+ cells isolated from GSI-nonresponder CML patients. **(c)** Overexpression of P-crkl in CD34+ CML cells treated with GSI. CD34+ cells from five CML patients in chronic phase were cultured in the presence of 10 μM GSI. The change in BCR-ABL activity was assessed by the FACS-based P-crkl assay. **(d)** P-crkl expression was measured by mean fluorescence intensity (MFI) units in each condition. MFI of P-crkl in GSI-treated CD34+ cells were compared to a no-drug control in each sample, and the percentage of increase in P-crkl was calculated. Data shown here represent the mean of six CML samples (**p<0.*01*).

To determine whether GSI could induce inhibition of Notch signalling in CD34+ CML primary cells, the expression of *Hes1* was investigated in CD34+ CML primary cells at 72h post-GSI (10 μM) treatment. Results showed that primary CD34+ cells from four CML samples (CML-2, CML-3, CML-4, and CML-5) responded well to GSI treatment, which was evident by significant (*p*<0.05) downregulation of *Hes1* (Fig 5a). However, CD34+ cells from two CML samples (CML-1 and CML-6) showed resistance to GSI treatment (Fig 5b).

#### (iv) GSI increases the kinase activity of BCR-ABL in CD34+ CML primary cells

To further explore the cross-talk between Notch and BCR-ABL, the effect of the Notch inhibitor GSI on BCR-ABL activity was investigated in CD34+ CML primary cells. Cells were cultured in growth factor cocktail for 72h in the presence of 10 μM GSI before measuring BCR-ABL activity by P-crkl assay. To calculate the change in P-crkl expression, the mean fluorescence intensity (MFI) of GSI-treated and untreated CD34+ cells was first determined by subtracting the MFI of P-crkl–stained cells from the MFI of isotype control in each condition. The MFI of GSI-treated cells was then compared with the MFI of untreated cells, and the change in P-crkl expression was reported as a percentage.

Interestingly, FACS data showed that GSI treatment increased the P-crkl expression in CD34+ cells between 18%–42% as compared with the control group (Fig 5c). Importantly, an increase in the P-crkl expression was seen in all the CD34+ CML primary cells that showed downregulation of *Hes1* mRNA post-GSI treatment, suggesting that the increase in P-crkl expression is Notch dependent. The increase in P-crkl in CD34+ CML cells (n = 6) was statistically significant (*p*<0.01), as shown in Fig 5d.

### Evidence of cross-talk between Notch signalling and BCR-ABL in chronic myeloid leukaemia cell line model

#### (i) K562 cell line as a model for BCR-ABL and Notch cross-talk

**Fig 6:**
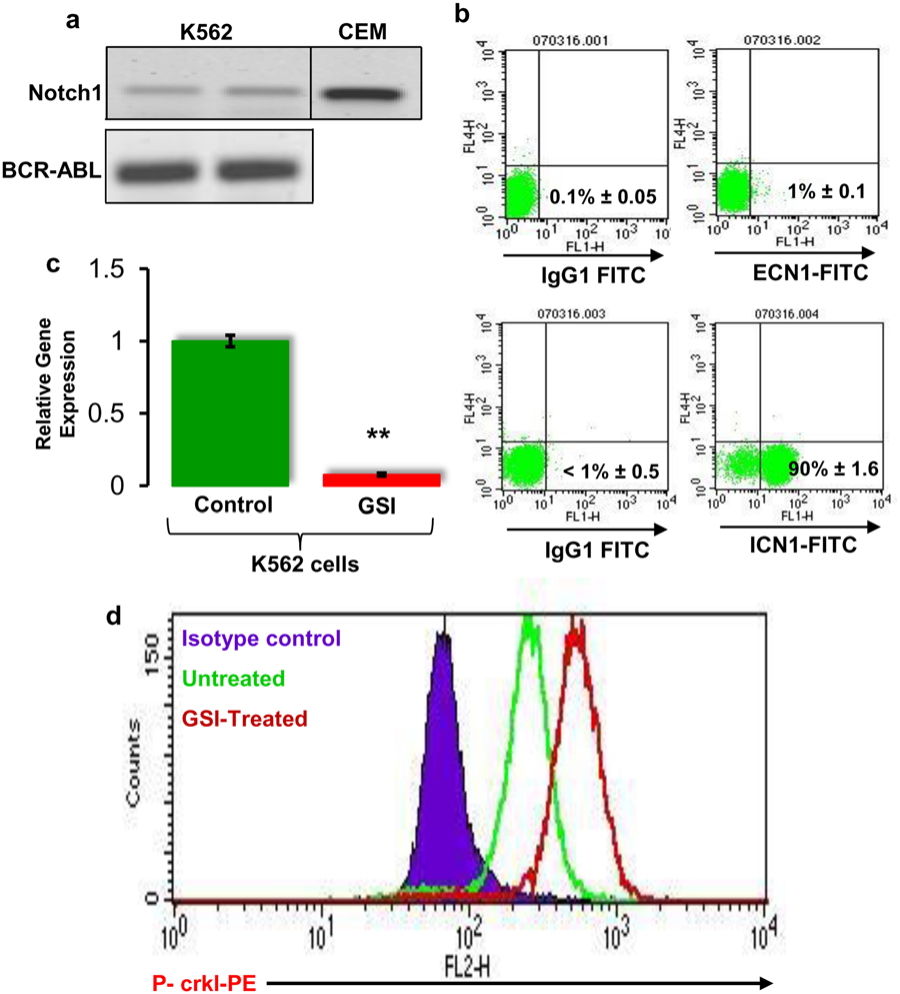
Expression of Notch1 in the K562 cell line. **(a)** Expression of *Notch1* in the K562 cell line model at transcriptional level. cDNA was prepared from K562 cells. The CEM cell line was used as a positive control for active Notch signalling. Transcript levels were measured by RT-PCR. RT-PCR products were resolved by agarose gel electrophoresis and visualised by Vistra Green (n = 3). **(b)** Analysis of Notch1 expression in K562 cells at protein level. Cells were stained with EA1 antibody to detect the extracellular domain of Notch1 (ECN1) and bTAN 20 antibody to detect intracellular domain of Notch1 (ICN1) using FACS. Appropriate isotype controls were used in each staining (n = 4). **(c)** Inhibition of Notch signalling by γ-seretase inhibitor (GSI) in K562 cells. The cDNA was prepared from cells treated with vehicle control (DMSO) and 10 μM GSI for 24h. Real-time PCR of the Notch target gene *Hes1* is shown (n = 5). ** = *p<0*.*01*. **(d)** The effect of Notch inhibition on BCR-ABL activity. K562 cells were cultured for 24h in the presence of GSI (10μM) and BCR-ABL activity was assessed by FACS based P-crkl assay (n = 4).

**Fig 7:**
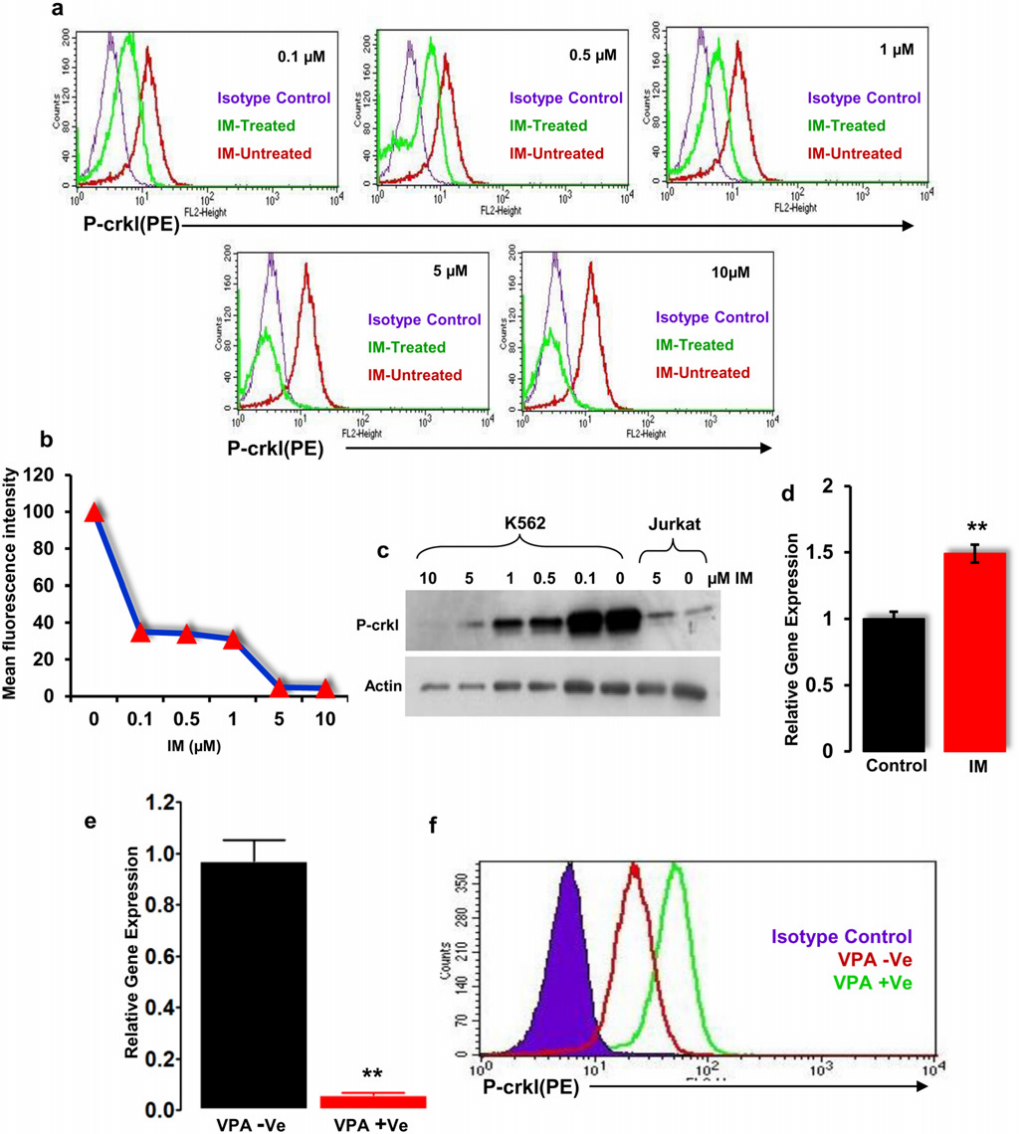
Cross-talk between Notch and BCR-ABL in the K562 and ALL-SIL cell line model. **(a)** Assessment of IM efficacy in K562 cells using P-crkl assay. K562 cells were cultured in increasing concentrations of IM (10, 5, 1, 0.5, and 0.1 μM) for 48h. P-crkl expression in cells treated with IM is shown. Data shown is from one experiment representative of three separate experiments (n = 4). **(b)** Dose-dependent effect of IM on the expression of P-crkl in K562 cells. P-crkl expression of IM treated and untreated K562 cells represented as mean fluorescence intensity (MFI) uing FACS as described in **(a)** (n = 4). **(c)** Concentration-dependent effect of IM on P-crkl protein. K562 and Jurkat cells cultured in increasing concentrations of IM (10, 5, 1, 0.5, and 0.1 μM) for 48h. P-crkl protein levels were measured by western blotting. **(d)** Expression of *Hes1* in K562 cells, 48h posttreatment. Notch target gene *Hes1* was assessed after 48h treatment with 10 μM IM (**p<0.*01*). **(e)** *Hes1* expression in K562 cells post valproic acid (VPA) treatment. K562 cells were treated with 4mM VPA for 72h and *Hes1* expression was measured by real-time PCR. Gene expression was normalised to the *GAPDH* (n = 3). Statistical significance was calculated using student t-test. (** = p <0.01). **(f)** Effect of VPA on BCR-ABL activity in K562 cells. K562 cells were treated with 4mM VPA for 72h and the activity of BCR-ABL was assessed by FACS analysis of P-crkl expression. (n = 3).

K562 cells are known to show high BCR-ABL activity, which can be inhibited by IM and expression of active Notch signalling [33]. Moreover, K562 cells express the Notch downstream target gene *Hes1* [14,16] at levels that can be inhibited by GSI; hence, it is a good cell line model to study the interaction between BCR-ABL and Notch. First, we confirmed the expression of BCR-ABL and *Notch1* in K562 cells (Fig 6a and 6b). We then validated for optimal P-crkl expression at different dilutions of a P-crkl antibody (S5a–S5c Fig) and at different passages (S5d–S5f Fig). GSI has been shown to inhibit Notch signalling in normal CD34+ cells and in T-ALL cell lines [22,34]. To investigate the effect on BCR-ABL activity following Notch inhibition, K562 cells were cultured with 10 μM GSI for 24h. The cells were then harvested, and an intracellular P-crkl expression was performed. *Hes1* expression was assessed by real-time PCR to confirm the inhibition of Notch activity. Real-time PCR results showed significant (*p*<0.01) downregulation of transcriptional target gene *Hes1* in the GSI-treated K562 cells (Fig 6c). The FACS analysis showed a dramatic increase in P-crkl expression in the GSI-treated cells compared with the control group (Fig 6d). To study the effect of IM-induced BCR-ABL inhibition on Notch signalling, cells were cultured in the presence of an increasing concentration of (0.1, 0.5, 1, 5 and 10 μM) IM for 48h. Following confirmation of significant dose-dependent P-crkl inhibition (Fig 7a–7c), real-time PCR was performed for *Hes1* expression. Results showed that *Hes1* was significantly (*p*<0.01) upregulated in K562 cells at 48h posttreatment with 10 μM IM (Fig 7d). Furthermore, we also evaluated the effects of histone deacetylase (HDAC) inhibitor valproic acid (VPA) in the K562 cell line model. Treatment of VPA significantly (*p* = 0.01) inhibited the Notch target gene *Hes1* (Fig 7e) and induced increased phosphorylation of crkl (Fig 7f) in the K562 cell line model.

#### (ii) ALL-SIL cell line as a model for ABL-Notch cross-talk

**Fig 8:**
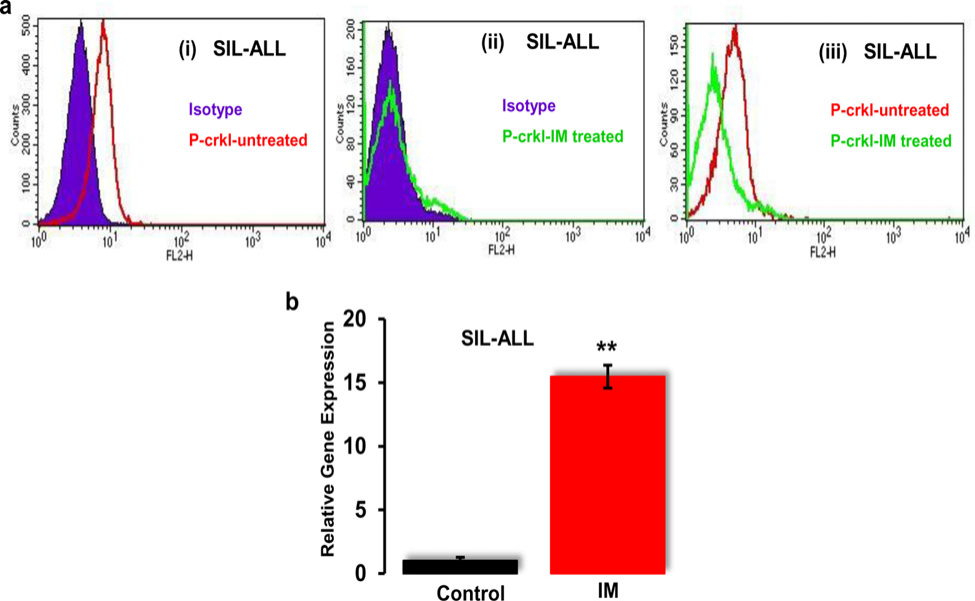
Cross-talk between Notch and BCR-ABL in the ALL-SIL cell line models. **(a)** Assessment of expression of P-crkl levels in the ALL-SIL cell line. (i) Cells were stained with P-crkl primary antibody and PE secondary antibody (red) and isotype control (blue). (ii) Expression of P-crkl in ALL-SIL cells after incubating the cells for 48h with 10 μM IM shown in green and isotype control in blue. (iii) P-crkl expression of IM-treated cells is shown in green as compared with untreated cells in red (n = 3). **(b)** Expression of *Hes1* at 48h posttreatment. Notch target gene *Hes1* mRNA was evaluated in ALL-SIL cells at 48h posttreatment with 10 μM IM using qRT-PCR (n = 4). ** = *p<0*.*01*, statistical significance difference between treated and untreated group.

We further assessed the ABL-Notch cross-talk in the ALL-SIL cell line model since the reported activity of ABL and Notch signalling in ALL-SIL cells [35,36] also makes this cell line a good *In Vitro* model to evaluate the cross-talk of ABL and Notch. The FACS-based P-ckrl assay showed the expression of P-crkl as a marker for the ABL kinase activity in ALL-SIL cells (Fig 8a). To study if the ABL activity can be switched off and to assess whether the P-crkl assay can be utilised as an IM sensitivity assay, the effect of IM was investigated on ALL-SIL cells.

ALL-SIL cells were cultured in the presence of 10 μM IM for 48h. The cells were then stained with P-crkl primary antibody and PE secondary antibody. Results showed that ABL kinase is active in ALL-SIL cells, and this activity is evident by the phosphorylation of P-crkl in the absence of IM. Treatment of ALL-SIL cells with IM resulted in clear reduction of P-crkl expression (Fig 8a). To further investigate the effect of ABL inhibition on Notch signalling, real-time PCR was performed to see the effect of IM on *Hes1*. Results showed that *Hes1* was significantly (*p*<0.01) upregulated in ALL-SIL cells at 48h posttreatment with 10 μM IM (Fig 8b).

### Validation of Notch-BCR-ABL cross-talk using CML CD34+ patients and NBM donor microarray datasets from GSE database system

**Fig 9:**
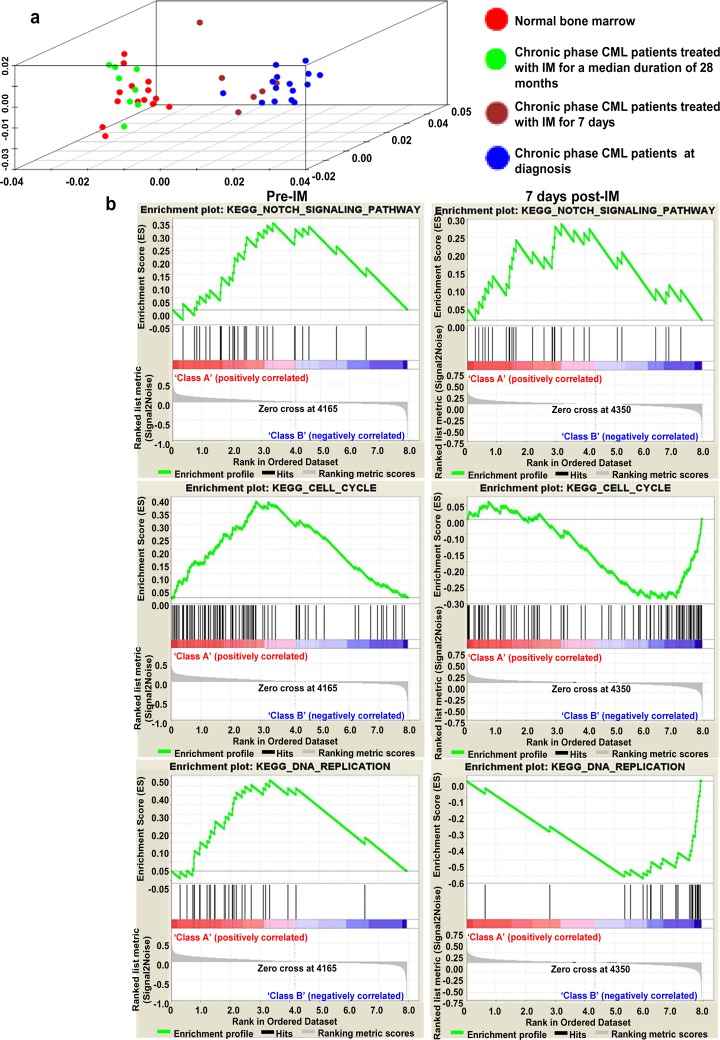
Analysis of microarray datasets from CML CD34+ patients and NBM donors. **(a)** Shows PCA plots of three gene expression datasets from the GSE library. Datasets comprise gene expression profiles of CD34+ haematopoietic stem cells from normal donors (n = 14), donors in chronic phase of CML (n = 15), donors treated with seven days of IM following a diagnosis of chronic phase CML (n = 6), and donors treated with IM for a median duration of 28 months (range 11–39 months) (n = 8). To identify the role of Notch signalling in chronic phase CML and following treatment with IM, gene set enrichment analysis was performed by comparing 14 bone marrow samples from normal subjects with 6 paired samples obtained from chronic phase CML patients before IM and after 7 days of IM treatment. **(b)** Shows regulation of genes involved in Notch signalling pre- and post-IM and its correlation with cell cycle pathways and DNA replication pathways.

To corroborate our *In Vitro* IM treatment data, we also performed a three-axis principal component analysis (PCA) on the three retrieved GSE microarray datasets. PCA analysis showed that patients treated with IM for a prolonged duration (28 months) clustered with NBM donors, and importantly, patients treated with IM for seven days showed a gene expression profile closely related to chronic phase CML (Fig 9a). However, at seven days post-IM treatment, some deviation in gene expression from chronic phase CML was observed, suggesting early changes in gene expression occur in CD34+ cells from chronic phase CML following a seven-day IM therapy.

**Fig 10:**
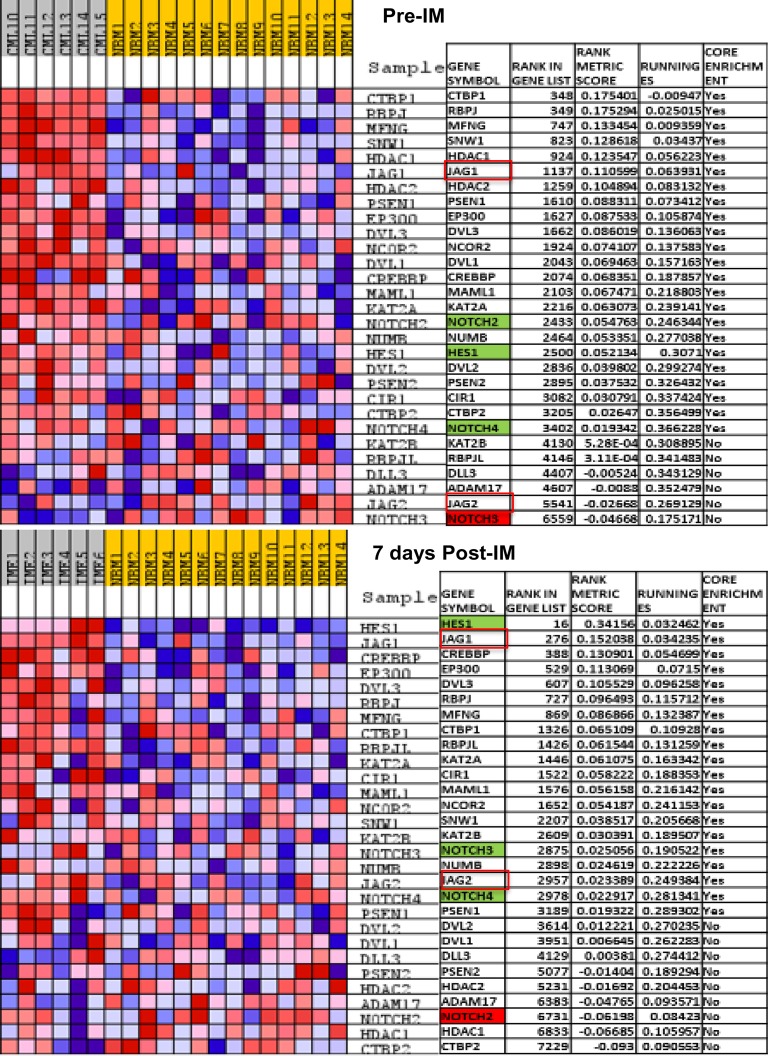
Blue-pink O'gram of altered Notch signalling is CML patient samples. **(a)** Shows Notch signalling pre-IM in CD34+ haematopoietic stem cells (HSCs) during chronic phase CML and **(b)** shows the effect of IM on Notch signalling in CD34+ HSCs during chronic phase CML.

To assess the role of Notch signalling in chronic phase CML and following BCR-ABL inhibition with IM therapy, we compared the gene expression profile of CD34+ cells from NBM donors with paired samples of CD34+ cells from chronic phase CML patients pre- and seven days post-IM therapy. Interestingly, we found that Notch signalling was upregulated both pre- and seven days post-IM therapy at false discovery rate (FDR) value of 0.005 and 0.1 as compared with NBM donors. Furthermore, in comparison with normal CD34+ cells from NBM, cell cycle and DNA replication pathways were significantly upregulated in CD34+ cells from chronic phase CML patients. Although these pathways were significantly downregulated in CD34+ cells from CML patients at seven days post-IM therapy, Notch signalling remained upregulated in both pre- and seven days post-IM therapy (Fig 9b). Similar to our *In Vitro* results, we observed that *Hes1* was significantly upregulated pre-IM therapy and further upregulated at seven days post-IM treatment. Importantly, we also observed that *JAG-1* and *JAG-2*, which are Notch target ligands, were also significantly upregulated post-IM (Fig 10). Thus, gene expression profile analysis from the retrieved datasets further corroborated our findings of activated Notch signalling during both hyperactivation and inhibition of BCR-ABL activity.

## Discussion

Notch signalling controls cell fate decisions, and stem cell renewal and differentiation [2,9]. The emerging evidence demonstrates the role of activated Notch signalling in haematopoietic malignancies [37]. Deregulation of Notch signalling is also implicated in the development of chronic B-cell lymphoid leukaemia [9]. Interestingly, activation of mutations of the Notch genes have been identified in more than 50% of human T-cell acute lymphoblastic leukaemia and in a subset of non-Hodgkin lymphomas [9]. Therefore, genetic or pharmacological manipulation of Notch signalling is a novel potential strategy for the treatment of many cancers. It is therefore important to understand how the genetic abnormality that causes cancer is related to Notch signalling. This study summarises the preclinical evidence, linking Notch signalling to BCR-ABL in chronic phase CML.

In the present study, the role of Notch signalling was evaluated in CML. *Notch1* and *Notch2* were significantly upregulated in the most primitive CD34+ Thy+ and total CD34+ subset in the chronic phase of CML. In particular, activation of Notch signalling in the CD34+ Thy+ subset indicates it may have a role in leukemic stem cell expansion similar to that of normal stem cells [38]. *Hes1* is upregulated in the CD34+ Thy+, CD34+ Thy-, and in the total CD34+ cell subsets. Moreover, the expression of *Notch1* in the CD34+ Thy+ and CD34+ CD38- cell subsets is interesting as these populations are enriched for leukemic stem cells in CML [39].

To understand the relationship between Notch and BCR-ABL, we used inhibitors of both Notch and ABL signalling and found that the Notch and BCR-ABL pathways antagonise each other in primary CD34+ cells isolated from chronic phase CML as well as in cell line models. Inhibition of BCR-ABL activity in primary CD34+ CML cells resulted in significant upregulation of *Hes1*. *Hes1* functions as a transcriptional repressor by interacting with histone deacetylase HDAC1 [40,41]. Interestingly, HDAC inhibitor VPA treatment in the K562 cell line model also resulted in the inhibition of Notch signalling/Hes-1 and overexpression of BCR-ABL activity, further confirming the antagonistic interaction between the Notch signalling pathway and BCR-ABL in the blastic phase of CML, which was seen with GSI treatment of K562 cells.

For the first time, these results show antagonistic interactions between the BCR-ABL and Notch signalling pathways in CD34+ chronic CML cells. Interestingly, a similar effect was not observed in IM-resistant samples. Since IM does not target Notch directly and does not influence γ-secretase activity *In Vitro* [42], these observations suggest that upregulation of *Hes1* was a BCR-ABL-mediated effect. Resistance of CML stem cells to IM has been reported [43]. The IM resistance observed here in total CD34+ cells from two CML patients could be due to either BCR-ABL mutations on the CD34+ cells or the presence of most primitive CD34+/CD38- cells at a high percentage in CML samples compared with IM-sensitive CML samples used in this study.

GSI treatment of CD34+ primary CML cells resulted in *Hes1* downregulation; however, it failed to show any effect in two CML samples (CML-1 and CML-6). Interestingly, CD34+ cells from those two CML patients who did not respond to GSI showed significant upregulation of *Hes1* when BCR-ABL activity was inhibited by IM. It is therefore possible that BCR-ABL may act as a Notch repressor, and its inhibition activates downstream proteins, which then activates the transcription factor RBP-J directly and results in Notch activation while bypassing the Notch receptor [44,45]. The other possibility is that GSI non-responding cells may have an activating mutation in the Notch pathway as explained in T-ALL [46]. In T-ALL cells, inhibition of aberrant Notch signalling by GSI leads to decreased proliferation [47] and increased sensitivity to apoptosis [22], suggesting that Notch contributes to the transformation of the cells. Therefore, activating Notch mutations may also occur in CML and may be responsible for the active Notch signalling in CML.

**Fig 11:**
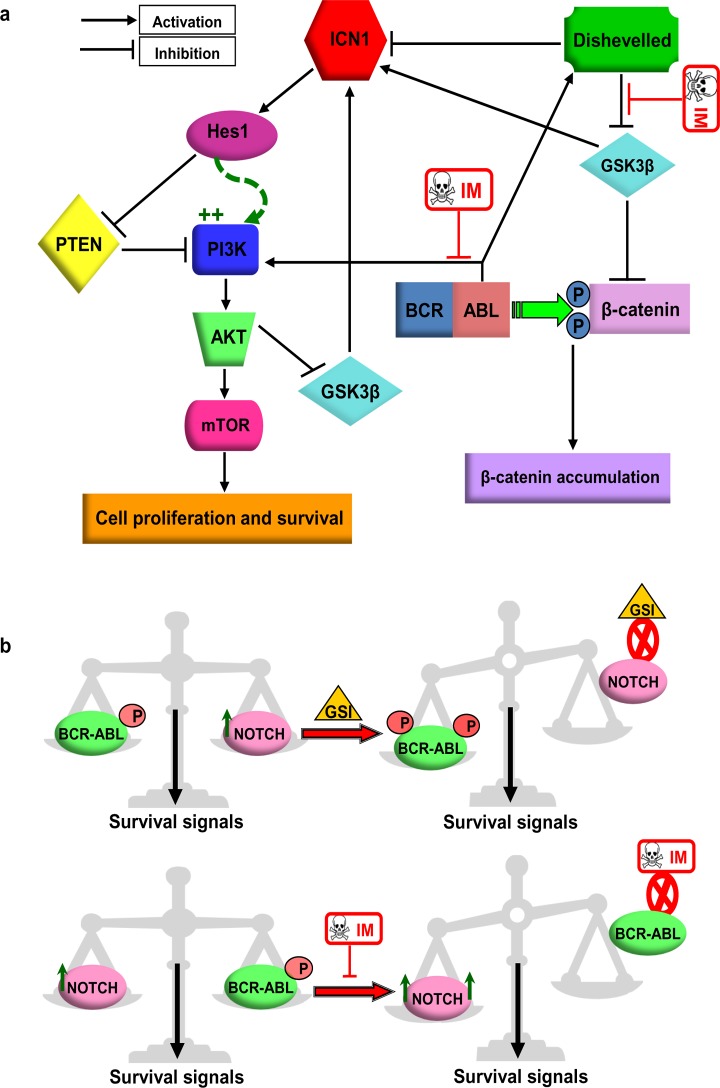
Schematic of proposed models. **(a)** Proposed model for Notch and BCR-ABL cross-talk in CML. Both BCR-ABL and Notch activate the PI3K/AKT-mTOR pathway that may trigger the survival and proliferation signals to CML cells. Blocking BCR-ABL kinase activity may not be sufficient to induce apoptosis as this may switch to survival signals to the PI3K pathway activated by Notch in CML cells. In this model, IM may be upregulating Notch by modulating Wnt component GSK3β and/or Dishevelled. IM-induced activation of GSK3β or inhibition of Dishevelled stabilises ICN in the cytoplasm, which in turn activates the PI3K/AKT-mTOR signalling by upregulation of *Hes1* which abolish the inhibitory effect of PTEN on PI3K pathway. **(b)** Cooperative model of activated Notch and BCR-ABL signalling in chronic phase CML. Both Notch and BCR-ABL are activated in chronic phase CML and may activate survival signalling pathways to inhibit apoptosis in CD34+ CML cells. *In vitro* inhibition of Notch by GSI-induced BCR-ABL activity to keep the same level of survival signals is required for CML cell. Treatment with IM leads to activation of Notch signalling to maintain the same level of survival signals needed by CML cells to inhibit apoptosis. The net effect is maintenance of balanced levels of survival signals that protect CD34+ CML cells from apoptosis in the chronic phase of CML (GSI: γ-secretase inhibitor, IM: imatinib mesylate).

Interestingly, *Hes1* transcription factor mediator of Notch signalling was shown to maintain hematopoietic and neuronal stem cell self-renewal as well as fetal T-cell and malignant CML progenitor immaturity [17,48]. Very recently, Sukanya *et al*. showed that CCN3 regulates Notch signalling in chronic myeloid leukaemia [49]. In the present study, supported by *In Silico* analyses, we have shown for the first time the direct antagonistic effect of Notch/*Hes-1* and BCR-ABL, and we have shown that inhibition of Notch signalling directly suppresses the overexpression of *Hes-1* in CML primary cells, which provides a rationale for the development of combinational therapeutic strategies targeting Notch and BCR-ABL in chronic phase CML. In T-ALL, Notch positively regulates the phosphatidylinositol 3-kinase (PI3K)/AKT pathway [50]. Furthermore, it has been shown that *Notch1* induces upregulation of the PI3K-AKT pathway via *Hes1*, which negatively controls the expression of PTEN [51]. In the present study *Hes1* was up-regulated in the ALL-SIL T-ALL cell line, a cell line that has constitutive Notch and ABL kinase activities, following the exposure to IM as demonstrated. It is possible that *Hes1* upregulation following IM exposure in CML cells may also activate the PI3K/AKT pathway and confer anti-apoptotic signals to CML cells regardless of the BCR-ABL repressed activity (Fig 11a). However, further work is required to investigate the activation of the PI3K pathway by Notch signalling, as reported in T-ALL, in CML cells. Although IM has been shown to inhibit BCR-ABL activity in CD34+ chronic phase CML cells, only a mild increase in apoptosis was demonstrated in these cells [33]. Moreover, it has been shown that IM treatment activated the PI3K/ Akt/ mammalian target of rapamycin (mTor) anti-apoptotic pathway in chronic phase CML patients as well as in BCR-ABL+ Lama cells [52] and proposed that the IM-induced compensatory PI3K-Akt/mTor activation may represent a novel mechanism for the persistence of BCR/ABL-positive cells in IM treated CML patients [52]. The IM induced activation of Notch signalling in the present study may suggest that, blocking the BCR-ABL activity by IM is may not enough to switch off the PI3K/AKT/mTor anti-apoptotic activity. It is also possible that the antagonistic effects between Notch and BCR-ABL signalling seen in this study may also involve other mechanism such as Wnt signalling pathway [12,53]. Importantly, haematopoietic progenitor cells have been reported to secrete Wnt [54] and it is possible that this pathway may therefore be active in CML. IM has been shown to inhibit Wnt signalling in CML cells [55] and in the murine myeloid progenitor cell line 32Dcl3 [56] in a way that may involve inhibition of Dishevelled and activation of GSK3β, both of which are key players in the canonical Wnt signalling pathway. Dishevelled has been reported to bind to Notch and down-regulate Notch signalling in *Drosophila* [57]. In contrast, it has been shown in cell line models that GSK3β positively modulates Notch signalling by protecting the intracellular domain of Notch1 (ICN1) from proteasome degradation [58]. Taken together, we suggest that, IM may activate Notch signalling by modulating the Wnt components Dishevelled and GSK3β (Fig 11a) or may activate Notch signalling by blocking the inhibitory action of BCR-ABL on its downstream substrate GSK3β.

Based on our results, we propose a cooperative model for Notch-BCR-ABL activity. Coexistence of activated Notch and BCR-ABL *In Vivo* in chronic phase CML suggests a cooperative interaction between the two signalling pathways and that both BCR-ABL and Notch signalling are equally critical for CML cell survival and resistance to apoptosis (Fig 11b). However, one should bear in mind that in the present study, we used pharmacological inhibitors to study the antagonistic effect of Notch and BCR-ABL instead of siRNA approach. The main difference between the two approaches is that RNA interference-mediated silencing removes the target mRNA from the cell whereas pharmacological inhibition only blocks the function of a protein, but the protein still is present. This might have implications—for example, the drug-inhibited protein may lack a certain activity but may still interact with some binding partners or assemble into macromolecular complexes and may also have many off-target effects. Nonetheless, our future study will be focused to further corroborate these findings using an in-depth RNAi approach in a large group of CD34+ CML patients.

In conclusion, preclinical data presented here strongly suggest a link between Notch and BCR-ABL in CML. Microarray dataset analysis revealed that inhibition of BCR-ABL with IM led to significant upregulation of *Hes1*, *JAG-1*, and *JAG-2*. This change in Notch signalling profile post-IM therapy clearly shows a response to IM. Therefore, we suggest that assessment of Notch signalling pre- and post-IM therapy in chronic phase CML patients may prove to be a useful biomarker in determining the primary IM resistance. Furthermore, our results suggest that blocking of Notch signalling by GSI, or using potential anti-Notch pharmacological small molecule inhibitors such as PF-03084014 [59], RO4929097 [60,61], GSI-953 [62], and anti-Notch mAb [63] along with IM may have a role in the treatment of CML. In particular, targeting Notch and BCR-ABL simultaneously may prove superior to tyrosine kinase monotherapy in advanced CML disease, and thus, such a combined approach should be explored.

## Supporting Information

S1 Fig Immunoreactivity of ECN1 and ICN1 antibodies. For the evaluation of ECN1 and ICN1 expression using western blotting, total HEK293 cell lysates were separated on 8% SDS-PAGE, transferred to nitrocellulose and probed with: **(a)**, EA1 antibody, which detects extracellular Notch1 (ECN1) and **(b)** b-tan20 antibody, which detects intracellular Notch1 (ICN1). Lanes (1) untransfected cells, (2&3) full-length human-Nothc1 transfected and (4) ICN transfected cells. (1&2)15g (3&4) 40g of cell lysates. The arrows indicate the full-length Notch1 (~300 kDa), the ECN1 (~180 kDa) and the ICN1 (~120 kDa). **(c)** Evaluation of ECN1 and ICN1 expression in HEK293 cells. HEK293 cells were transfected with full-length Notch1 and stained with ECN1 (EA1) and ICN1 (b-tan20) antibodies.
(TIF)

S2 Fig Notch expression profile on the CD34+ progenitors in primary CML cells. Mononuclear cells from CML samples were stained with CD34, specific myeloid lineage markers and the anti-extra cellular Notch1 (ECN1). **Panel a** shows co-staining with CD34 and isotype control (IgG1) and **panel b** shows co-staining with CD34 and ECN1 antibody (EA1) (n = 3).
(TIF)

S3 Fig Panel a, Expression of Notch1 in the CD34+ Thy+ primitive stem cell sub-populations. Mononuclear cells from CML samples (n = 3) were co-stained with ECN1 and the stem cell marker thy-1. The upper panel shows the gating strategy where only cells positive for both CD34 and Thy-1 used in the analysis of Notch1 expression. The lower panel shows that Notch1 is expressed in the primitive CD34+ thy+ population in CML primary cells (n = 3). IgG1 was used as an isotype control. **Panel b-c,** CD34 gating strategy and the Notch expression in CML primitive stem cell CD34+ CD38- cell subset in CML. Mononuclear cells from CML samples were co-stained with CD34 and anti Notch1 antibody (EA1) and the stem cell markers CD34, and CD38-. **Panel b** show the CD34 gating strategy used in all FACS plots in this study. The expression of Notch1 in the total CD34+ population in CML is shown in the right hand side of **panel b** as compared to the isotype control IgG1 in the middle plot. **Panel c** shows the Notch1 expression in the primitive CD34+ CD38- cell subset, enriched for stem cells (n = 3).
(TIF)

S4 Fig Evaluation of P-crKl expression in primary chronic myeloid leukaemia (CML) cells. Mononuclear cells from primary CML cells were cultured for 24h in cytokines cocktail before being fixed and stained with P-crkl primary antibody and either PE **(a)** or FITC **(b)** conjugated anti-rabbit secondary antibodies. K562 cells were used as positive control. Cells stained with P-crkl PE are shown in red, whereas unstained cells and isotype control are shown in blue and green respectively (**a**). The P-crkl FITC stained cells are depicted in green and isotype control in red (**b**) (n = 3).
(TIF)

S5 Fig Intracellular P-crkl staining in K562 cell line model. **(a)** Validation of P-crkl staining using FACS. Background staining on unfixed-unstained cells is shown in blue, fixed-unstained cells shown in green and P-crkl expression after fixation is shown in red. **(b)** Analysis of effect of secondary antibody staining in P-crkl assay. **(c)** Titration of the primary P-crkl antibody. (**d-f)** Effect of cell passage number on the expression of P-crkl in K562 cell line. K562 cells were taken out from liquid nitrogen and maintained in culture for 12 weeks. Cells were passaged every 4 days and P-crkl expression was assessed by FACS every two-weeks. (**d)** Passage 4–16; (**e)** passage 20, and **(f)** >24 passage.
(TIF)

S1 Table List of antibodies used in this study. (DOCX)

S2 Table Oligonucleotide sequences and annealing temperatures used in this study for PCR. (DOCX)

S3 Table List of Primers used in this study for real time PCR. (DOCX)
