# Supplementary figures and images for: Potential Role of Notch Signalling in CD34+ Chronic Myeloid Leukaemia Cells: Cross-Talk between Notch and BCR-ABL
Source: PLoS One. 2015 Apr 7;10(4):e0123016. doi: 10.1371/journal.pone.0123016 (PMC4388554; doi:10.1371/journal.pone.0123016)

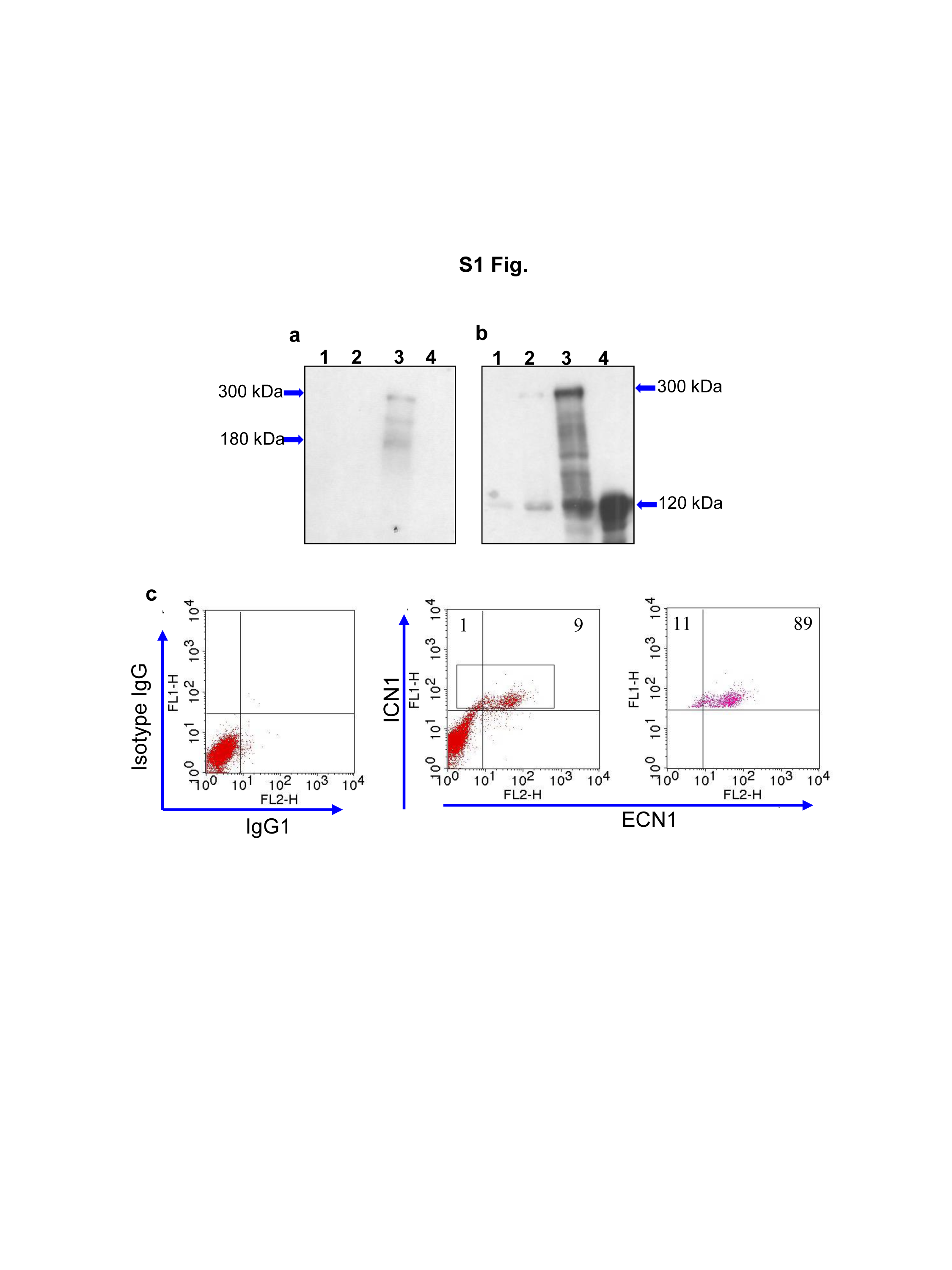

Supplement: S1 Fig — For the evaluation of ECN1 and ICN1 expression using western blotting, total HEK293 cell lysates were separated on 8% SDS-PAGE, transferred to nitrocellulose and probed with: (a), EA1 antibody, which detects extracellular Notch1 (ECN1) and (b) b-tan20 antibody, which detects intracellular Notch1 (ICN1). Lanes (1) untransfected cells, (2&3) full-length human-Nothc1 transfected and (4) ICN transfected cells. (1&2)15g (3&4) 40g of cell lysates. The arrows indicate the full-length Notch1 (~300 kDa), the ECN1 (~180 kDa) and the ICN1 (~120 kDa). (c) Evaluation of ECN1 and ICN1 expression in HEK293 cells. HEK293 cells were transfected with full-length Notch1 and stained with ECN1 (EA1) and ICN1 (b-tan20) antibodies. (TIF) [file pone.0123016.s001.tif]

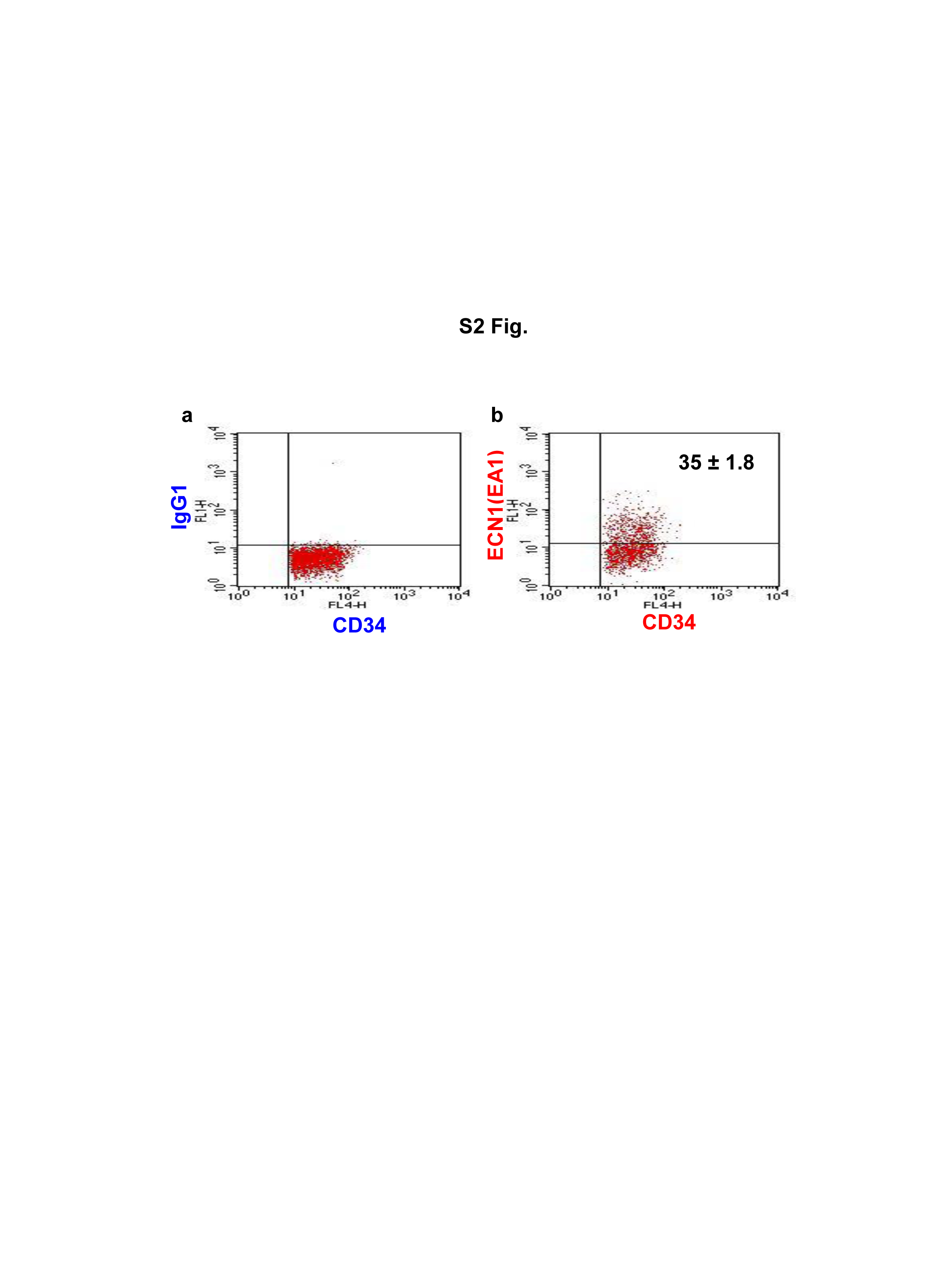

Supplement: S2 Fig — Mononuclear cells from CML samples were stained with CD34, specific myeloid lineage markers and the anti-extra cellular Notch1 (ECN1). Panel a shows co-staining with CD34 and isotype control (IgG1) and panel b shows co-staining with CD34 and ECN1 antibody (EA1) (n = 3). (TIF) [file pone.0123016.s002.tif]

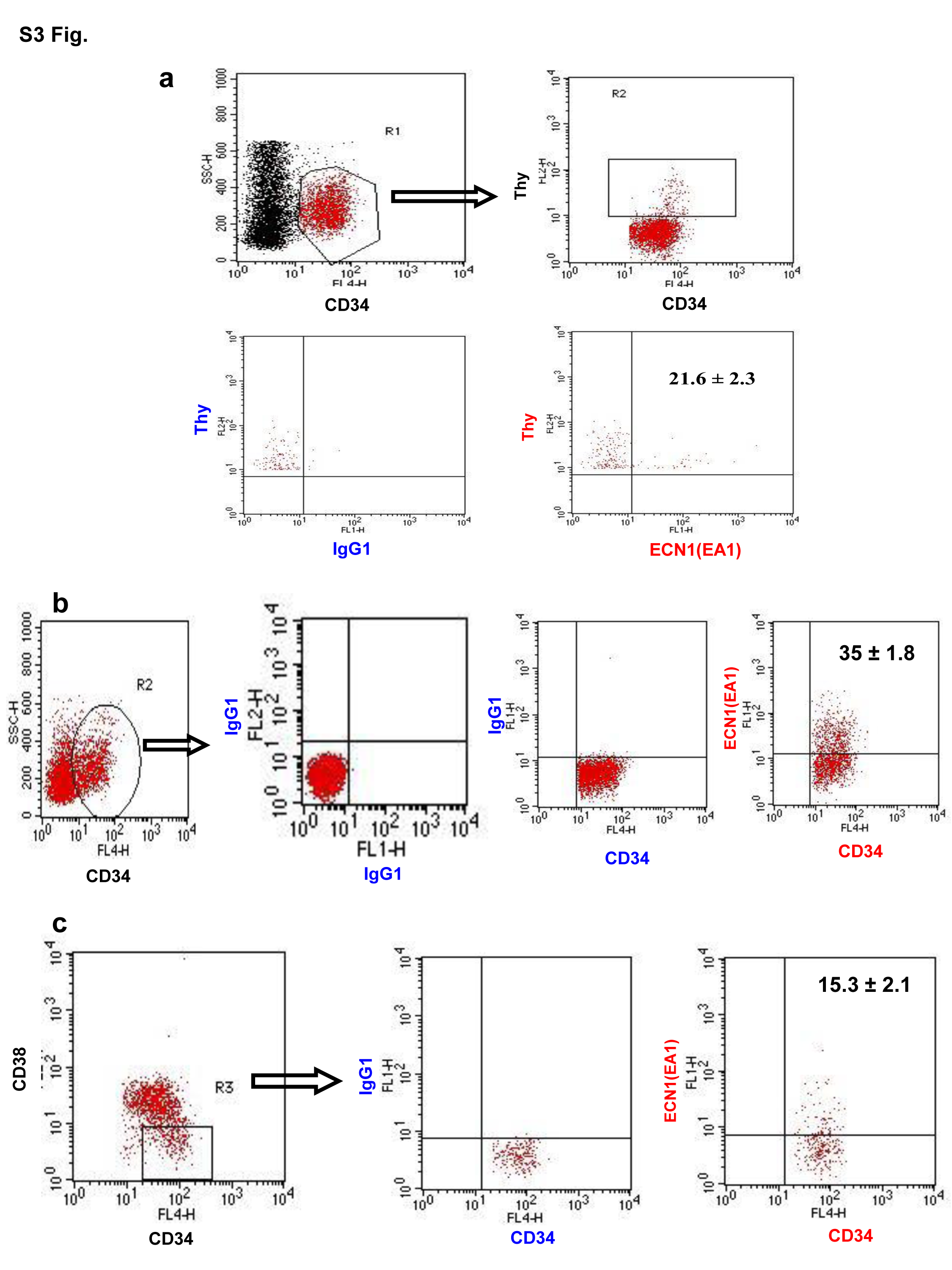

Supplement: S3 Fig — Mononuclear cells from CML samples (n = 3) were co-stained with ECN1 and the stem cell marker thy-1. The upper panel shows the gating strategy where only cells positive for both CD34 and Thy-1 used in the analysis of Notch1 expression. The lower panel shows that Notch1 is expressed in the primitive CD34+ thy+ population in CML primary cells (n = 3). IgG1 was used as an isotype control. Panel b-c, CD34 gating strategy and the Notch expression in CML primitive stem cell CD34+ CD38- cell subset in CML. Mononuclear cells from CML samples were co-stained with CD34 and anti Notch1 antibody (EA1) and the stem cell markers CD34, and CD38-. Panel b show the CD34 gating strategy used in all FACS plots in this study. The expression of Notch1 in the total CD34+ population in CML is shown in the right hand side of panel b as compared to the isotype control IgG1 in the middle plot. Panel c shows the Notch1 expression in the primitive CD34+ CD38- cell subset, enriched for stem cells (n = 3). (TIF) [file pone.0123016.s003.tif]

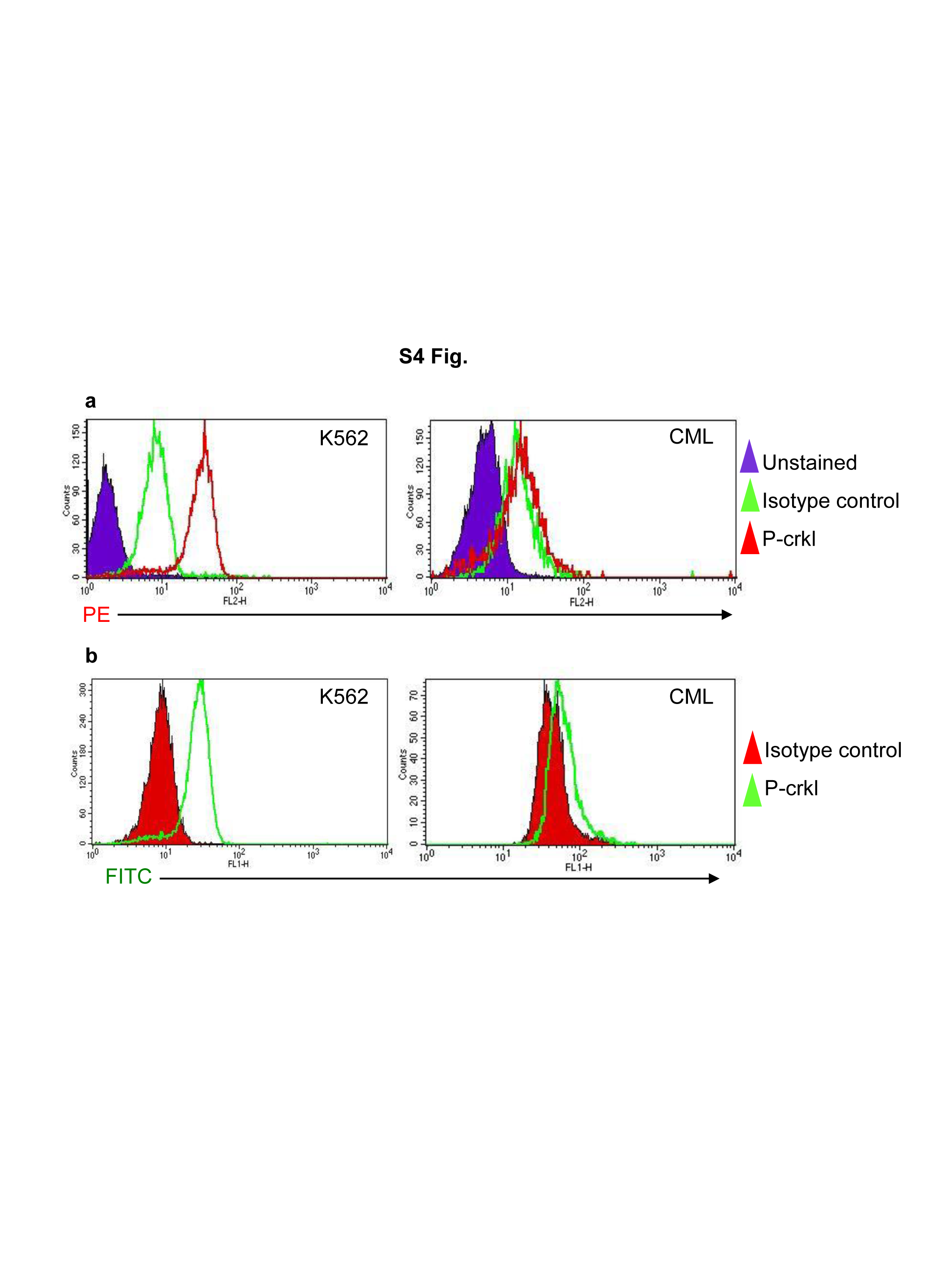

Supplement: S4 Fig — Mononuclear cells from primary CML cells were cultured for 24h in cytokines cocktail before being fixed and stained with P-crkl primary antibody and either PE (a) or FITC (b) conjugated anti-rabbit secondary antibodies. K562 cells were used as positive control. Cells stained with P-crkl PE are shown in red, whereas unstained cells and isotype control are shown in blue and green respectively (a). The P-crkl FITC stained cells are depicted in green and isotype control in red (b) (n = 3). (TIF) [file pone.0123016.s004.tif]

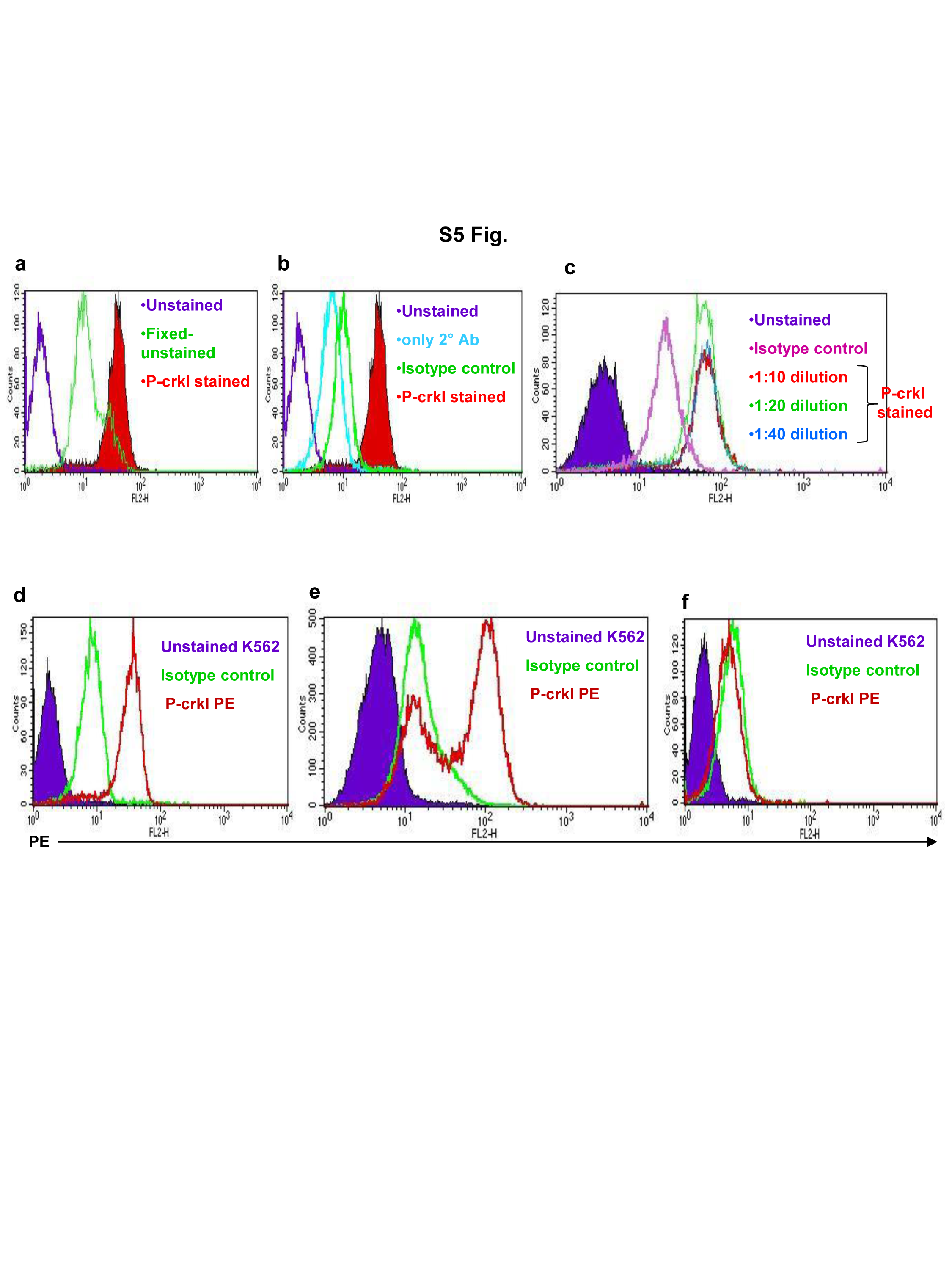

Supplement: S5 Fig — (a) Validation of P-crkl staining using FACS. Background staining on unfixed-unstained cells is shown in blue, fixed-unstained cells shown in green and P-crkl expression after fixation is shown in red. (b) Analysis of effect of secondary antibody staining in P-crkl assay. (c) Titration of the primary P-crkl antibody. (d-f) Effect of cell passage number on the expression of P-crkl in K562 cell line. K562 cells were taken out from liquid nitrogen and maintained in culture for 12 weeks. Cells were passaged every 4 days and P-crkl expression was assessed by FACS every two-weeks. (d) Passage 4–16; (e) passage 20, and (f) >24 passage. (TIF) [file pone.0123016.s005.tif]
