# Supplementary material for: Potential Role of Notch Signalling in CD34+ Chronic Myeloid Leukaemia Cells: Cross-Talk between Notch and BCR-ABL
Source: PLoS One. 2015 Apr 7;10(4):e0123016. doi: 10.1371/journal.pone.0123016 (PMC4388554; doi:10.1371/journal.pone.0123016)
Supplement: S1 Table — (DOCX) [file pone.0123016.s006.docx]

**S1Table**. List of antibodies used in this study.

| **Monoclonal Ab** | **Fluorochrome conjugate** | **Target/ lineage specificity** | **Dilution** | **Supplier** |
| --- | --- | --- | --- | --- |
| **b-TAN20** | FITC | Ubiquitous | 1:5 | DSHB |
| **P-crkl** | FITC or PE | ABL+ HCs | 1:40 | Cell signaling |
| **CD90**  **(Thy-1)** | PE | Primitive HCs | 1:20 | pharmingen |
| **CD34** | APC | Primitive HCs | 1:20 | BD Bioscience |
| **CD38**  **CD15** | FITC | T, B and CD34+ committed cells | 1:50 | BD Bioscience |
| **CD14**  **CD15** | FITC | Myeloid cells | 1:50 | BD Bioscience |
| **CD15** | FITC | Myeloid cells | 1:25 | BD Bioscience |
| **CD16** | FITC | Myeloid cells | 1:50 | BD Bioscience |
| **CD33** | PE | Myeloid cells | 1:50 | BD Bioscience |
| **CD7** | FITC | T cells | 1:25 | BD Bioscience |
| **CD3** | FITC | T cells | 1:25 | BD Bioscience |
| **CD19** | FITC | B cells | 1:20 | BD Bioscience |
| **CD45** | PE | Pan HCs | 1:50 | BD Bioscience |
| **IgG1** | FITC | Control | 1:20 | BD Bioscience |
| **IgG1** | PE | Control | 1:20 | BD Bioscience |
