# Supplementary material for: Potential Role of Notch Signalling in CD34+ Chronic Myeloid Leukaemia Cells: Cross-Talk between Notch and BCR-ABL
Source: PLoS One. 2015 Apr 7;10(4):e0123016. doi: 10.1371/journal.pone.0123016 (PMC4388554; doi:10.1371/journal.pone.0123016)
Supplement: S2 Table — (DOCX) [file pone.0123016.s007.docx]

**S2 Table.** Oligonucleotide primer sequences and annealing temperatures used in this study for PCR.

| **Gene name** | **Primer** | **Primer Sequence 5’ – 3’** | **Length**  **(bp)** | **Amplicon**  **Size**  **(bp)** | **T ºC** |
| --- | --- | --- | --- | --- | --- |
| **Notch1** | hN1F | GTGAGGGACGTCAGACTTGG | 20 | 166 | 58 ºC |
|  | hN1R | AACATCTTGGGACGCATCTG | 20 |  |  |
| **Notch2** | hN2F | AAAGCATCTGTCAAATAGGAAAC | 23 | 205 | 58 ºC |
|  | hN2R | TAAGGAATGTTACAAACCAATCA | 23 |  |  |
| **Notch3** | hN3F | CAAGCTGGATTCTGTGTACCTAGT | 24 | 202 | 56 ºC |
|  | hN3R | CCCCAGCAAGGCTATGGAACA | 21 |  |  |
| **Notch4** | hN4F | ATATTTATTGGGCACCTACTAATG | 23 | 166 | 58 ºC |
|  | hN4R | ATAGCAATAGCAGTGGCTAGAAG | 23 |  |  |
| **Hes1** | Hes1F | GTATTAAGTGACTGACCATG | 20 | 140 | 54 ºC |
|  | Hes1R | TCAAACATCTTTGGCATCAC | 20 |  |  |
| **Herp1** | Herp1F | TCATTTCTCTACTGTGTGGAG | 21 | 155 | 60 ºC |
|  | Herp1R | GTGGTATGTAAAGACTCTTGC | 21 |  |  |
| **Herp2** | Herp2F | CTAATTTTCCTGGGACTGCC | 20 | 216 | 60 ºC |
|  | Herp2R | TCAAACCCAGTTCAGTGGAG | 20 |  |  |
| **GAPDH** | GAPDHF | CCAGCAAGAGCACAAGAGGAAGAG | 24 | 180 | 56 ºC |
|  | GAPDHR | AGCACAGGGATACTTTATTAGATG | 24 |  |  |
| **BCR-ABL** | BCR-ABL F | TCCACTCAGCCACTGGATTTAA | 22 | 100 | 60 ºC |
|  | BCR-ABL R | TGAGGCTCAAAGTCAGATGCTACT | 24 |  |  |
