# Supplementary material for: Potential Role of Notch Signalling in CD34+ Chronic Myeloid Leukaemia Cells: Cross-Talk between Notch and BCR-ABL
Source: PLoS One. 2015 Apr 7;10(4):e0123016. doi: 10.1371/journal.pone.0123016 (PMC4388554; doi:10.1371/journal.pone.0123016)
Supplement: S3 Table — (DOCX) [file pone.0123016.s008.docx]

**S3 Table.** List of Primers and Probes used in this study for real time PCR.

| **Target** | **Primer** | **Primer Sequence 5’ – 3’** | **Annealing**  **Temp °C** |
| --- | --- | --- | --- |
| **Notch1** | hN1F | TCCCCCGGCTCTACGG | 60 |
|  | hN1R | ACACAGTAAAAATCAACATCTTGGGAC |  |
|  | hN1TP | CCGCGTGGTGCCATCCCC |  |
| **Notch2** | hN2F | AGCCATAGCTGGTGACAAACAG | 60 |
|  | hN2R | CAACTACTTCGCATTTCCATTGG |  |
|  | hN2TP | AGGCACCTTGTCCCTGAGCAACC |  |
| **Hes1** | Hes1TF | GCCACCCCTCCTCCTAAACTC | 60 |
|  | Hes1TR | TCAAAGAGAAGGAGGCAAGGAAA |  |
|  | Hes1TP | CAACCCACCTCTCTTCCCTCCGGA |  |
